# A convolutional neural network-based deep learning approach for predicting surface chloride concentration of concrete in marine tidal zones

**DOI:** 10.1038/s41598-025-12035-1

**Published:** 2025-07-29

**Authors:** Mohamed Abdellatief, Mahmoud E. Abd-Elmaboud, Mohamed Mortagi, Ahmed M. Saqr

**Affiliations:** 1 Department of Civil Engineering, Higher Future Institute of Engineering and Technology in Mansoura, Mansoura, Egypt; 2 Irrigation and Hydraulics Department, Faculty of Engineering, Mansoura University, Mansoura, 35516 Egypt; 3 Structural Engineering Department, Faculty of Engineering, Mansoura University, Mansoura, 35516 Egypt; 4 Faculty of Engineering, Mansoura National University, Mansoura, Egypt

**Keywords:** Surface chloride concentration, Convolutional neural network, Deep learning, Tidal zones, Gaussian process regression, Sustainable development goals, Civil engineering, Marine chemistry, Structural materials

## Abstract

Chloride-induced corrosion is a major threat to the durability of reinforced concrete (RC) structures. This is especially critical in marine tidal zones, where surface chloride concentration (Cs) plays a key role in predicting chloride ingress using Fick’s second law. However, traditional assessment methods are time-consuming and impractical, necessitating advanced predictive models. This study developed a deep learning-based framework utilizing a convolutional neural network (CNN) trained on 284 samples with 11 critical features related to material composition and environmental conditions. The CNN’s performance was benchmarked against four machine learning (ML) models: stepwise linear regression (SLR), support vector machine (SVM), Gaussian process regression (GPR), and random forest (RF). Results demonstrated CNN’s superiority, achieving a coefficient of determination (R^2^) = 0.849 and a lower root mean square error (RMSE) = 0.18%, outperforming conventional models. Shapley additive explanation (SHAP) analysis revealed exposure time, water content, and fine aggregate as the most critical factors influencing Cs predictions. The findings highlighted the importance of material composition and environmental exposure in optimizing concrete mix designs to mitigate chloride ingress in tidal zones. This research can enhance durability assessment, proactive maintenance strategies, and service life estimation of RC structures in harsh marine environments. Furthermore, it can contribute to the sustainable development goals (SDGs) by promoting resilient infrastructure, sustainable construction practices, and improved climate adaptation strategies. By integrating deep learning in durability assessments, this study can provide a scalable, efficient solution for optimizing maintenance planning and reducing premature failures in coastal RC structures, ultimately extending their service life.

**Supplementary Information:**

The online version contains supplementary material available at 10.1038/s41598-025-12035-1.

## Introduction

Reinforced concrete (RC) structures, including sea-crossing bridges, ports, docks, and coastal edifices, are extensively erected in marine settings but are susceptible to chloride-induced corrosion from seawater and airborne chlorides^1–3^. Chloride ions penetrate the concrete matrix, destroying the protective passive oxide film on reinforcing steel, initiating corrosion, and causing cracking, spalling, and reduced load-carrying capacity^4–8^. This degradation frequently causes early failure of RC structures, resulting in safety hazards, engineering failures, and significant economic losses^4,9,10^. A tragic example is the Florida coastal building collapse, where extensively corroded reinforcement was found^11^. Protective treatments, such as silane/siloxane, silicon resin, and fluoropolymer coatings, are applied to mitigate chloride ingress^12–16^. In addition to tidal zone exposure, seawater and airborne chlorides from marine aerosols pose significant risks, underscoring the importance of durability-focused design and service life prediction models that comprehensively account for exposure conditions and deterioration mechanisms^12,17–19^. These developments are essential for guaranteeing the durability and safety of RC structures in severe marine conditions^20–23^.

**Fig. 1:**
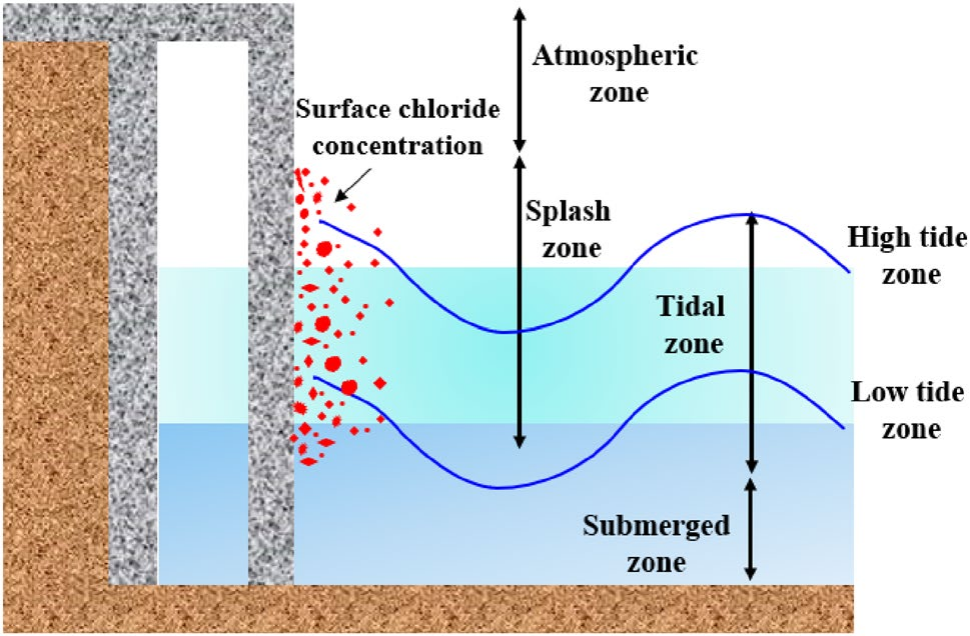
Different exposure zones of marine structure.

Marine ecosystems are often classified into four main zones: atmospheric, tidal, splash, and submerged. Each zone is defined by unique mechanisms and intensities of chloride exposure, as illustrated in Fig. 1^24,25^. Among these zones, the tidal zone presents unique challenges in terms of chloride transport due to periodic immersion and exposure to seawater, making it a critical area for investigation. In the tidal zone, the transmission of chloride into the RC is governed not only by diffusion but also by absorption. This combination of mechanisms significantly influences the chloride ingress rate and the subsequent corrosion of reinforcing steel. Recent research on surface chloride concentration (Cs) has increasingly focused on developing non-destructive techniques and methodologies to accurately estimate apparent Cs from test results, marking a significant advancement in the assessment of RC structures. For example, Lehner^24^ employed a method involving chloride captured on a plate surface, commonly used in steel bridge corrosion analysis, to determine Cs in concrete, while Andrade^25^ and Sadowski^26^ investigated the influence of the convection zone on calculating apparent Cs. Given the complexities of on-site Cs measurement, numerous prediction models have been developed, and recommended parameter values for related models have been proposed. Additionally, the European technical guide DuraCrete^27^ introduced a model for quantitatively calculating Cs, which has since been referenced and enhanced in standards like LNEC E465 and the Standard for Water Transport Design (JTS 153–2015). Likewise, ACI 138 and the JSCE Guide offer recommended Cs values tailored to typical environmental conditions. Beyond these standards, researchers have proposed various forecasting methods based on regression analyses and experimental data, capturing the effects of multiple factors on Cs. It has been widely reported that the Cs value rises with the water-cement ratio^1,12,28,29^ and the fly ash content decreases with the slag content^1^. Cs value also increases with exposure duration, and this time-dependent relationship is often modeled using logarithmic, power, or square root functions^27,30^. Environmental factors such as wind speed and proximity to the shore significantly affect Cs. Broadly, prediction models for Cs are categorized into material methods^31^time-dependent methods^32^and environmental methods^3^based on the parameters considered during their development. Lately, Cai et al.^1^ developed conventional machine learning (ML) methods to forecast Cs of concrete in three zones (splash, tidal, and submerged), which is vital for durability design in marine environments. Using records of 642 experimental data, including material and environmental conditions, they trained five ML methods, i.e., multilayer perceptron (MLP), support vector machine (SVM), Gaussian process regression (GPR), stepwise linear regression (SLR), and random forest (RF), as well as an ensemble model. The ensemble model, combining predictions from RF, MLP, and SVM, outperformed the standalone models.

In this study, the focus is exclusively on the tidal zone, where chloride is introduced through absorption during the wetting phases, and then diffused during drying phases. This process results in a more complex chloride transport mechanism compared to the submerged zone, where concrete remains continuously saturated^33,34^. The tidal zone undergoes periodic wetting and drying, which facilitates chloride ingress into the concrete via capillary absorption, as well as diffusion. The variation in exposure conditions, such as wave action and periodic submersion, further complicates the transport behavior of chloride. Fick’s Law of diffusion, which is generally utilized to model chloride ingress, can still be applied in the tidal zone, but with the inclusion of absorption as an additional factor. This law provides a foundational approach to understanding chloride penetration over time. The modified form of the solution accounts for both diffusion and absorption as follows^1,10^:

1 $$\:\text{C}\left(\text{x},\text{t}\right)={\text{C}}_{0\:}+\left({\text{C}}_{\text{s}\:}+{\text{C}}_{0\:}\right)\times\:\:\left[1-\text{e}\text{r}\text{f}\left(\frac{\text{x}}{2\sqrt{\text{D}\times\:\text{t}}}\right)\right]$$

where, C(x, t) is the concentration at a depth x from the concrete surface following an exposure duration t. C_0_ signifies the initial bulk concentration, and D represents the apparent chloride diffusion coefficient.

The error function (erf) mathematically characterizes the diffusion process, depicting the temporal dispersion of chloride within the concrete. The principal difficulty in forecasting chloride infiltration in the tidal zone stems from the interplay between diffusion and absorption, which fluctuates considerably based on environmental factors such as tidal frequency, wave exposure, and the length of wetting and drying cycles. Comprehending these interactions is crucial for precisely forecasting the long-term performance and longevity of RC structures in tidal regions. Subsequent studies in this domain must concentrate on enhancing the models for chloride intrusion in tidal regions, considering the cyclical wetting and drying effects and their impact on diffusion and absorption rates. This approach enables us to accurately forecast the effects of chloride exposure on the durability of RC structures in tidal marine settings^20–23,35,36^.

## Research significance and objectives

This study aimed to advance the prediction of Cs in RC structures located in tidal zones using a deep learning-based approach. The primary objective was to enhance the durability design of RC by providing accurate and reliable Cs estimates. To achieve this, the study leveraged the ability of convolutional neural networks (CNNs) to model complex and non-linear interactions among multiple influencing factors. By focusing on material properties and environmental conditions, the approach sought to capture the real-world variability that affects chloride ingress. The study also compared the CNN model’s performance with traditional machine learning techniques to establish its superiority in predictive accuracy^37^. In addition, a sensitivity analysis was conducted to identify the most influential features affecting Cs. This analysis can help clarify how different variables, such as exposure duration, ambient conditions, and concrete composition, interact to influence chloride accumulation. By offering a transparent and interpretable prediction model, the study can contribute to more informed material selection and optimized structural design. Ultimately, the proposed approach can support the development of durable marine infrastructure by minimizing chloride-induced damage and reducing long-term maintenance costs to support sustainable development goals (SDGs)^38,39^.

## Methodology

**Fig. 2:**
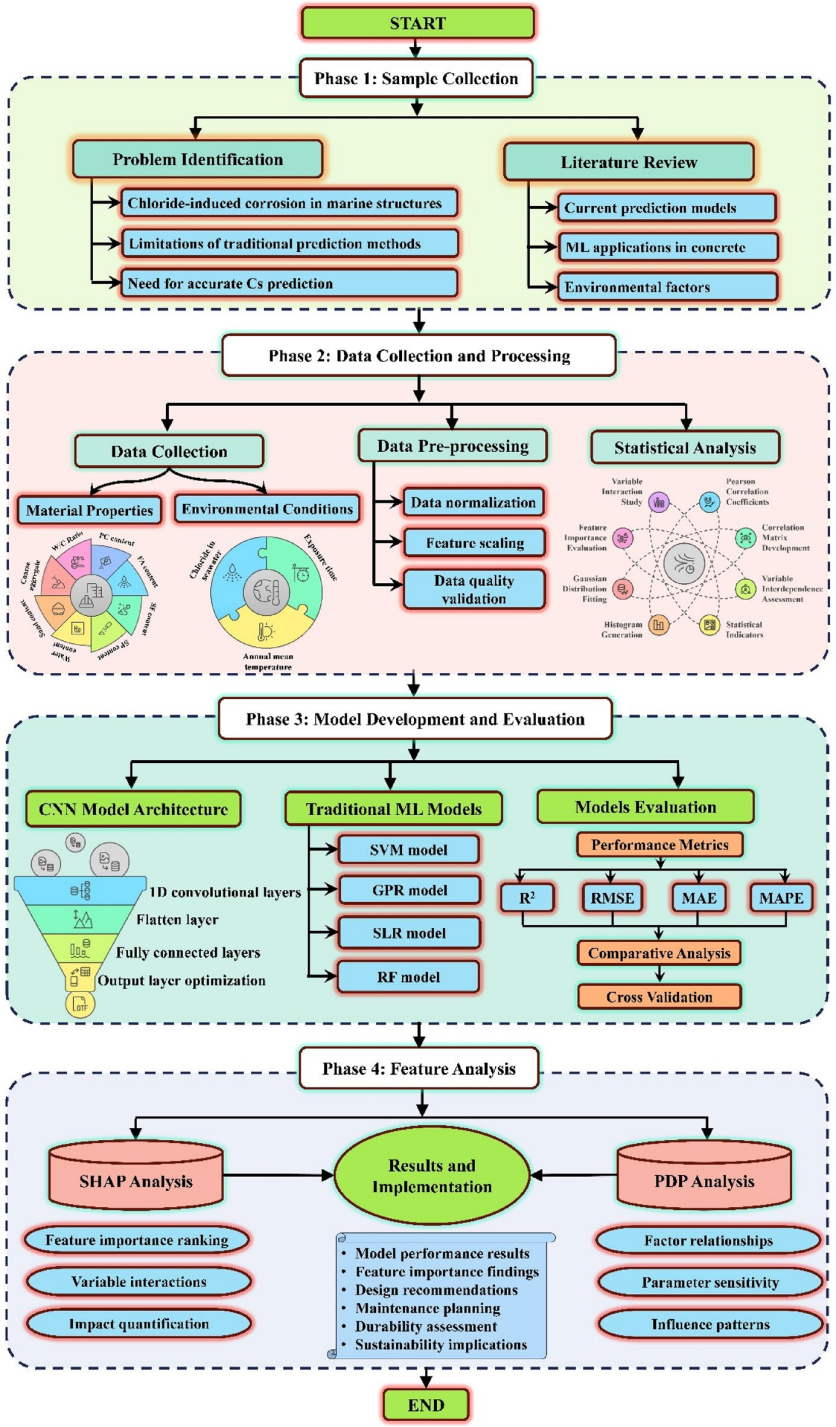
Visual diagram for study workflow.

In concrete science^18,19,40^ML workflows generally follow six phases, i.e., problem identification, data acquisition, data pre-processing, development of the model, evaluation of the model, and deployment of the model, which were systematically applied in this study to predict Cs in marine concrete using a deep learning-based approach^41,42^. The problem was first defined by recognizing the need for accurate predictions of Cs to enhance durability design and service life estimation of RC structures exposed to tidal zones. A dataset consisting of 284 field exposure records was subsequently compiled, encompassing 11 essential features related to the output variable (Cs values). The collected data underwent precise pre-processing to ensure quality and consistency, including normalization and encoding where necessary before being used for training the predictive models. During the model development phase, a deep CNN was employed to learn non-linear complex interconnections between the features and the target output, Cs. The CNN was benchmarked against four traditional ML models to assess its performance. During the model evaluation phase, the performance of all models was assessed using key statistical metrics, including coefficient of determination (R^2^), mean squared error (MSE), and root mean squared error (RMSE)^41,43^. Following evaluation, the best-performing model was deployed to predict Cs for concrete mixtures not included in the training dataset. These predictions were validated against existing empirical models to ensure reliability. Additionally, a sensitivity analysis was conducted to quantify the effect of each feature on Cs, offering insights into the interplay of independent variables in determining Cs. A corresponding flowchart (Fig. 2) illustrates the ML workflow adopted in the study, demonstrating its systematic approach for predicting Cs of concrete in marine tidal zones.

### Data collection

The current investigation leveraged records, including 284 experimental datasets collected from previous articles, providing a robust foundation for predicting Cs in concrete structures located in tidal zones. The dataset consolidated field data from various published references, ensuring a diverse and reliable basis for analysis (Table 1). The data were sourced from numerous peer-reviewed studies, including^1,15,24,25,31,44–46^. This metadata was crucial for understanding experimental configurations and enabling comparisons among various research studies^47–49^. The variables were carefully selected to comprehensively represent the factors influencing Cs. These include eight concrete mixture design variables, i.e., portland cement, fly ash, silica fume, superplasticizer, water content, fine aggregate, coarse aggregate, and water-cement ratio, along with two environmental variables, annual mean temperature, and chloride concentration in seawater, as well as a single exposure time variable measured in years. Together, these 11 input variables were chosen for their relevance to Cs and their ability to reflect both material composition and environmental influences. This dataset can serve as the input for the ML framework, enabling the deep CNN to model the non-linear complex interconnection between Cs and independent parameters.

**Fig. 3:**
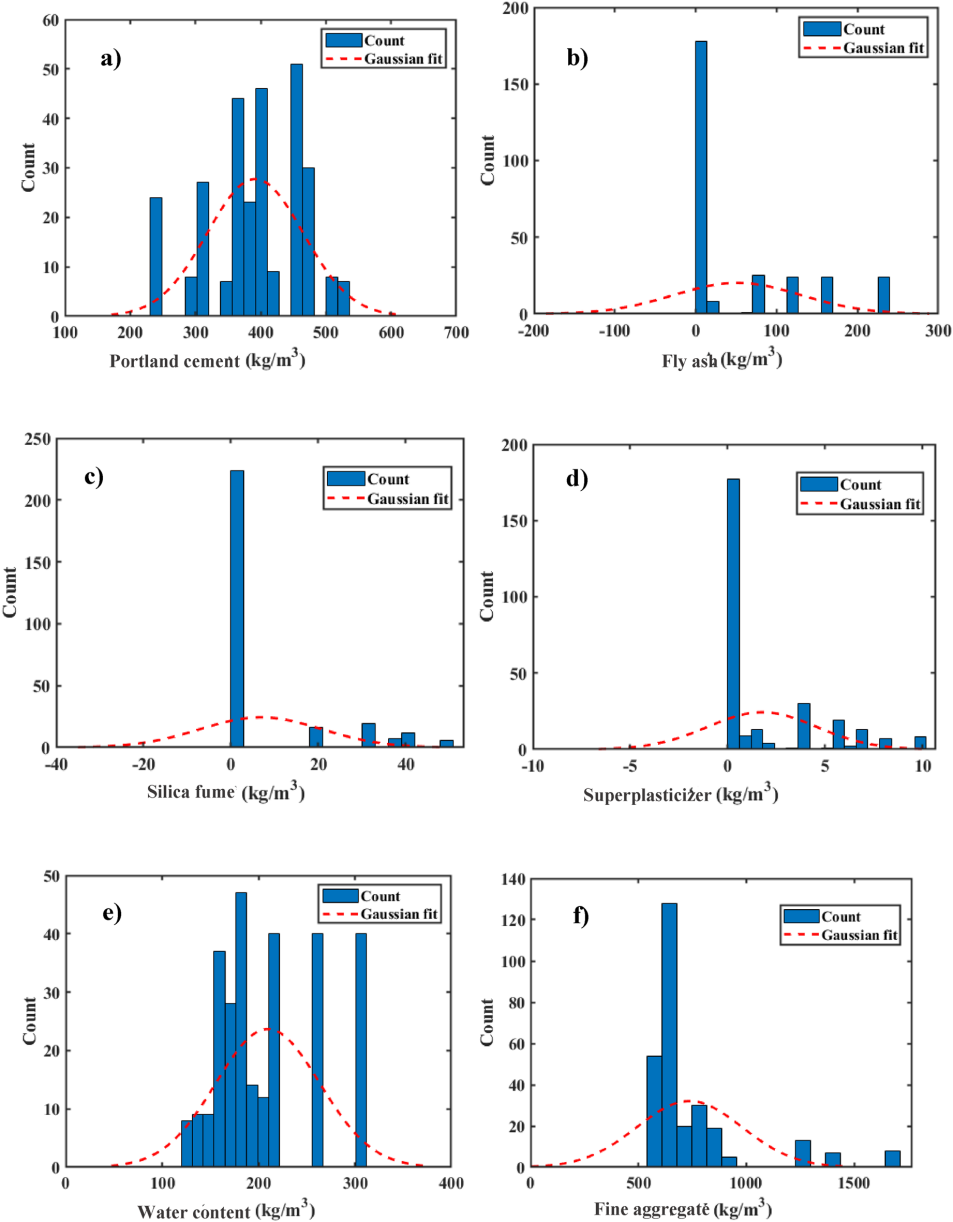

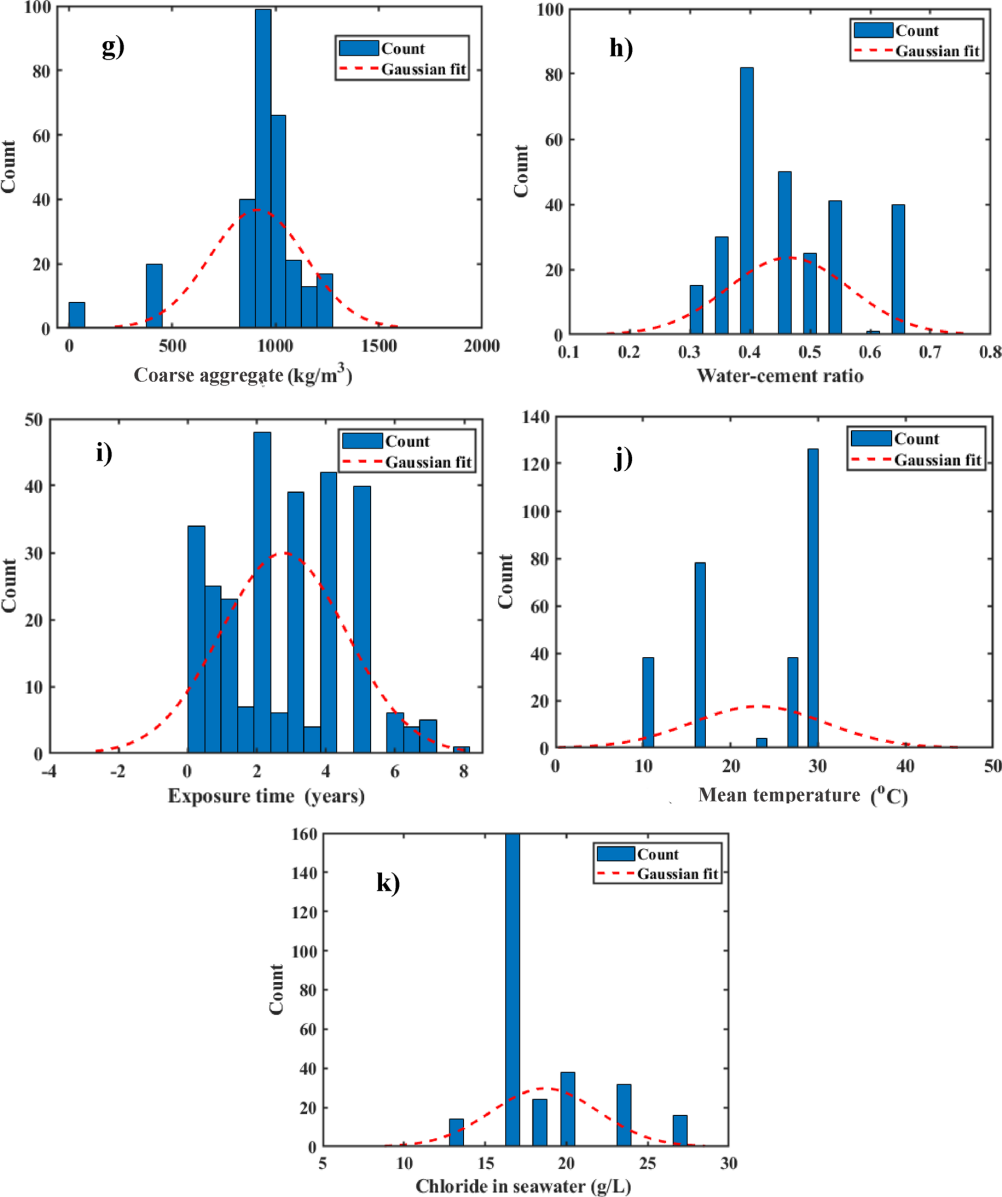
Samples of variable distribution alongside the Gaussian distribution curve.

Figure 3 presents the distribution of both independent and dependent variables, with the vertical axis representing the frequency of records for each parameter value and the horizontal axis showing the value range for each variable. The figure also includes a Gaussian fit to the relevant data. For instance, the diagram for Portland cement indicated that a large proportion of the Cs records fell within the 200–500 kg/m^3^ range. Additionally, the distribution of input variables was shown, with water content following a nearly normal distribution, while fine aggregate content displayed a left-skewed distribution, with most samples concentrated in the 500–1000 kg/m^3^ range. The coarse aggregate data was mostly clustered around 1000 kg/m^3^. Overall, Fig. 3 provides significant insights into the distribution patterns of both independent and dependent variables, hence boosting data comprehension.

**Table 1:** Statistical factors and key features of surface chloride concentration (Cs) records.

Factors	Descriptive indicators						
	Average	Median	Standard deviation	Minimum	Maximum	Variance	Skewness
Chloride concentration (% wt. Concrete)	0.85	0.92	0.39	0.08	0.15	0.20	– 0.11
Portland cement (kg/m^3^)	391.71	400.00	73.51	239.00	5385.25	3055.77	– 0.44
Fly ash (kg/m^3^)	51.48	0.00	78.95	0.00	6211.34	169.70	1.29
Silica fume (kg/m^3^)	6.86	0.00	13.96	0.00	194.14	316.02	1.76
Superplasticizer (kg/m^3^)	1.77	0.00	2.80	0.00	7.79	4.85	1.48
Water content (kg/m^3^)	209.63	190.00	54.07	127.50	2913.06	1876.86	0.69
Fine aggregate (kg/m^3^)	736.55	639.00	243.72	552.00	59191.80	34541.23	2.51
Coarse aggregate (kg/m^3^)	912.85	955.50	231.23	0.00	53281.28	38963.55	– 2.18
Water-cement ratio (kg/m^3^)	0.46	0.45	0.10	0.30	0.01	0.01	0.52
Exposure time (year)	2.77	2.67	1.82	0.16	3.29	2.85	0.39
Mean temperature (°C)	23.20	27.50	7.64	10.00	58.22	34.65	– 0.54
Chloride in seawater (g/L)	18.67	17.00	3.28	13.00	10.75	18.51	1.21

**Fig. 4:**
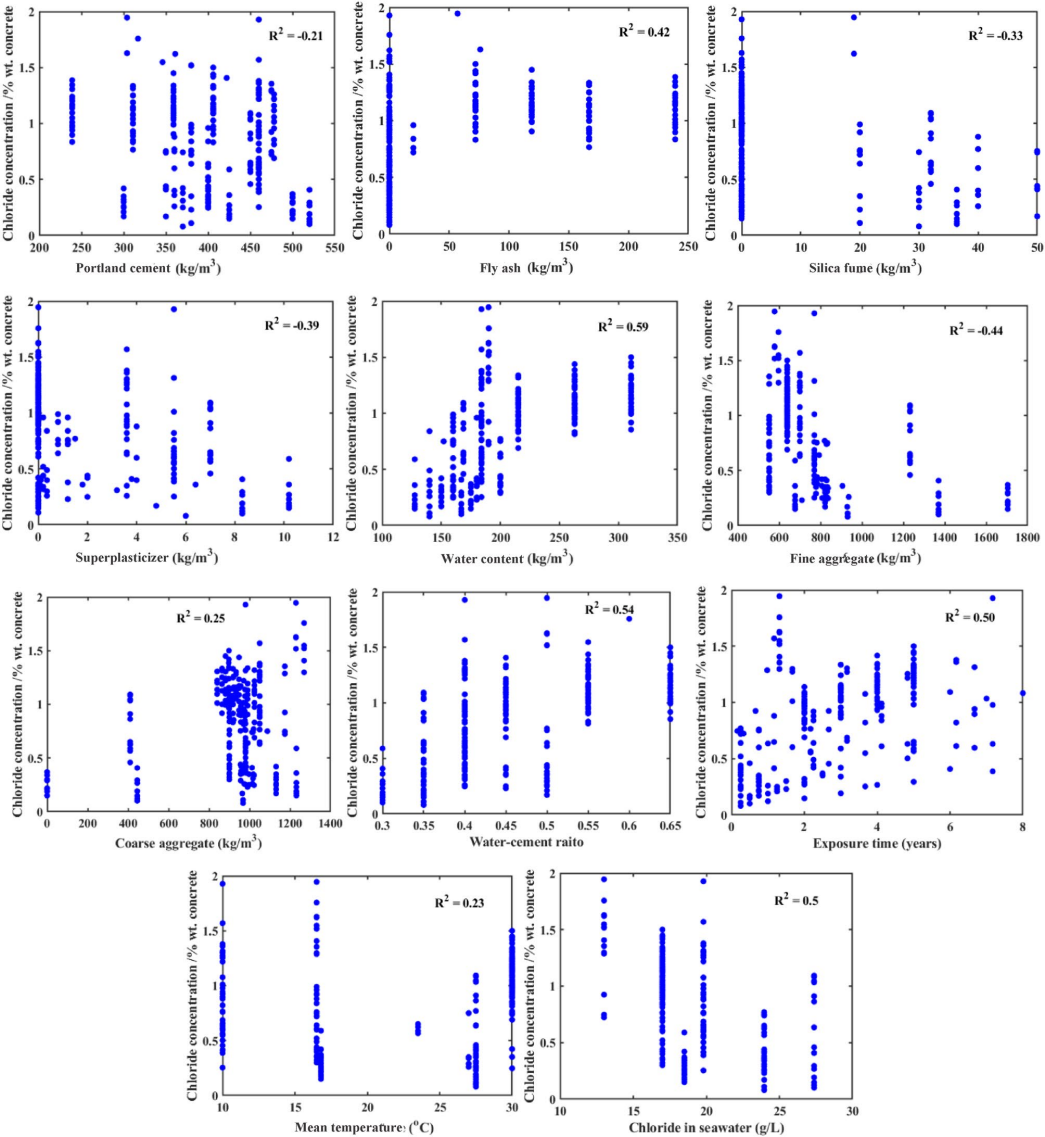
Scatter plots illustrating the relationships between each individual input variable and Cs. The R^2^ reflects the strength of the linear association and is used here for exploratory analysis to identify potentially influential variables prior to model development.

Figure 4 presents the relationships between Cs and the chosen 11 input variables to measure the strength and direction of these correlations. The analysis revealed expected negative correlations between fine aggregate, superplasticizer, and Cs. Conversely, a positive correlation between water content, fly ash, and Cs was observed, providing insight into the interplay of these variables. This visualization indicated how each independent variable influenced Cs and aided in identifying key factors critical to developing an accurate model^41^.

To further assess the influence of the chosen input parameters on Cs, the Pearson correlation coefficient (PCC) was applied as a widely recognized method for measuring linear relationships. PCC is computed as the ratio of the covariance to the product of the standard deviations of two variables (x & y), as expressed in Eq. (2). This approach can enable researchers to evaluate the significance of each variable’s relationship with Cs, aiding in the identification and prioritization of the most impactful factors for model development^50^.

2 $$\:\text{P}\text{C}\text{C}=\:\frac{\text{c}\text{o}\text{v}(\text{x},\:\text{y})}{{{\upsigma\:}}_{x}{{\upsigma\:}}_{y}}=\frac{\sum\:({\text{x}}_{i}-\:\stackrel{-}{\text{x}})({\text{y}}_{i}-\:\stackrel{-}{\text{y}})}{\sqrt{\sum\:{({\text{x}}_{i}-\:\stackrel{-}{\text{x}})}^{2}}\sqrt{\sum\:{({\text{y}}_{i}-\:\stackrel{-}{\text{y}})}^{2}}}\:$$

where, $\:\stackrel{-}{\text{x}}$ and $\:\stackrel{-}{\text{y}}$ represent the means of the x and y datasets, respectively.

**Fig. 5:**
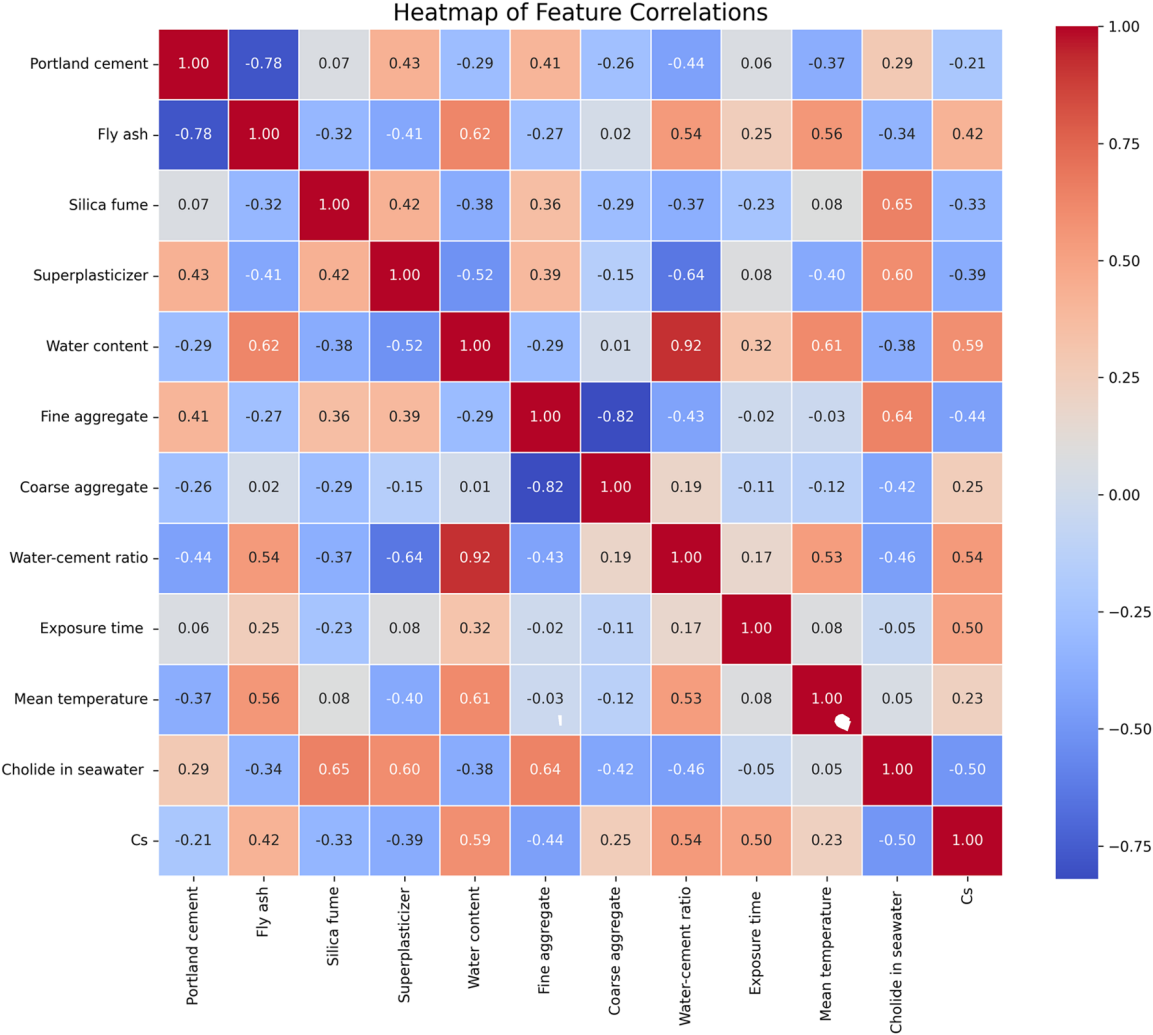
Pearson correlation coefficient **(**PCC) matrix of the chosen 11 input parameters and surface chloride concentration (Cs).

Figure 5 shows the PCC heatmap for the 11 input variables and Cs. Significant relationships were noted among all variables. In this investigation, exposure time, fine aggregate, water-cement ratio, superplasticizer, and water content were found to have a more significant impact on Cs compared to other parameters. Moreover, the findings indicated an absence of significant linear association among silica fume, fly ash, and Cs.

### Data splitting and performance assessment of machine learning (ML) models

Models for predicting Cs were developed using five distinct approaches: RF, SLR, SVM, GPR, and CNN. Each model requires optimization of specific factors to achieve optimal performance. The dataset was randomly divided into 80% for training and 20% for testing, following experimentation with various splitting ratios to assess the generalization error of the prediction models during the training phase^22^. A five-fold cross-validation procedure was employed for the regression techniques. The process involved randomly dividing the training set into five subsets or folds. The model was trained on four of these five folds, with the remaining fold used to evaluate the model’s error. The procedure was executed five times, with a distinct fold as the validation set each time, to guarantee a thorough assessment of the model’s efficacy^51^. The findings were subsequently averaged to yield a more dependable assessment of the model’s generalization capability. The average errors from all five folds were used to calculate the validation error. To quantitatively assess the forecasting performance of the CNN structure and other methods (using the testing data), four model accuracy metrics were employed, including coefficient of determination (R^2^), root mean square error (RMSE), mean absolute error (MAE), and mean absolute percentage error (MAPE). These metrics measure the cumulative error in the predicted Cs for the testing data compared to the experimental results^52^. The mathematical expressions for calculating these matrices are provided in Table 2, where y′ and y represent the forecasted and actual values, and n is the total number of records in the testing data.

**Table 2:** Assessment metrics of the proposed machine learning (ML) techniques.

Validation criteria	Formula	Eq. No.
R^2^	$\:1-\frac{{\sum\:}_{i}{\left({y}_{i}-{y}_{i}^{{\prime\:}}\right)}^{2}}{{\sum\:}_{i}{\left({y}_{i}-{\stackrel{-}{y}}_{i}\right)}^{2}}$	(3)
RMSE	$\:\sqrt{\frac{1}{n}\sum\:_{i=1}^{n}{({y}_{i}-{y}_{i}^{{\prime\:}})}^{2}}$	(4)
MAE	$\:\frac{1}{n}\sum\:_{i=1}^{n}\left|{y}_{i}-{y}_{i}^{{\prime\:}}\right|$	(5)
MAPE	$\:\frac{1}{n}\sum\:_{i=1}^{n}\left|\frac{{y}_{i}^{{\prime\:}}-{y}_{i}}{{\stackrel{-}{y}}_{i}}\right|$	(6)

Where, the coefficient of determination (R^2^), ranges from 0 to 1, with values closer to 1 indicating better model fit^53,54^. The model quality was evaluated using metrics such as root mean square error (RMSE), mean absolute error (MAE), and mean absolute percentage error (MAPE), with best-fit values approaching zero^52,55^. n is the total number of records in the testing data. y′ and y represent the forecasted and actual values, while $\:{\stackrel{-}{y}}_{i}\:$is the mean value of actual values.

## Machine learning (ML) models

In this investigation, we utilized deep learning techniques, including one-dimensional (1D) CNN versus conventional ML models, to predict the Cs in concrete structures, located in tidal zones.

### One-dimensional convolutional neural network (1D CNN)

**Fig. 6:**
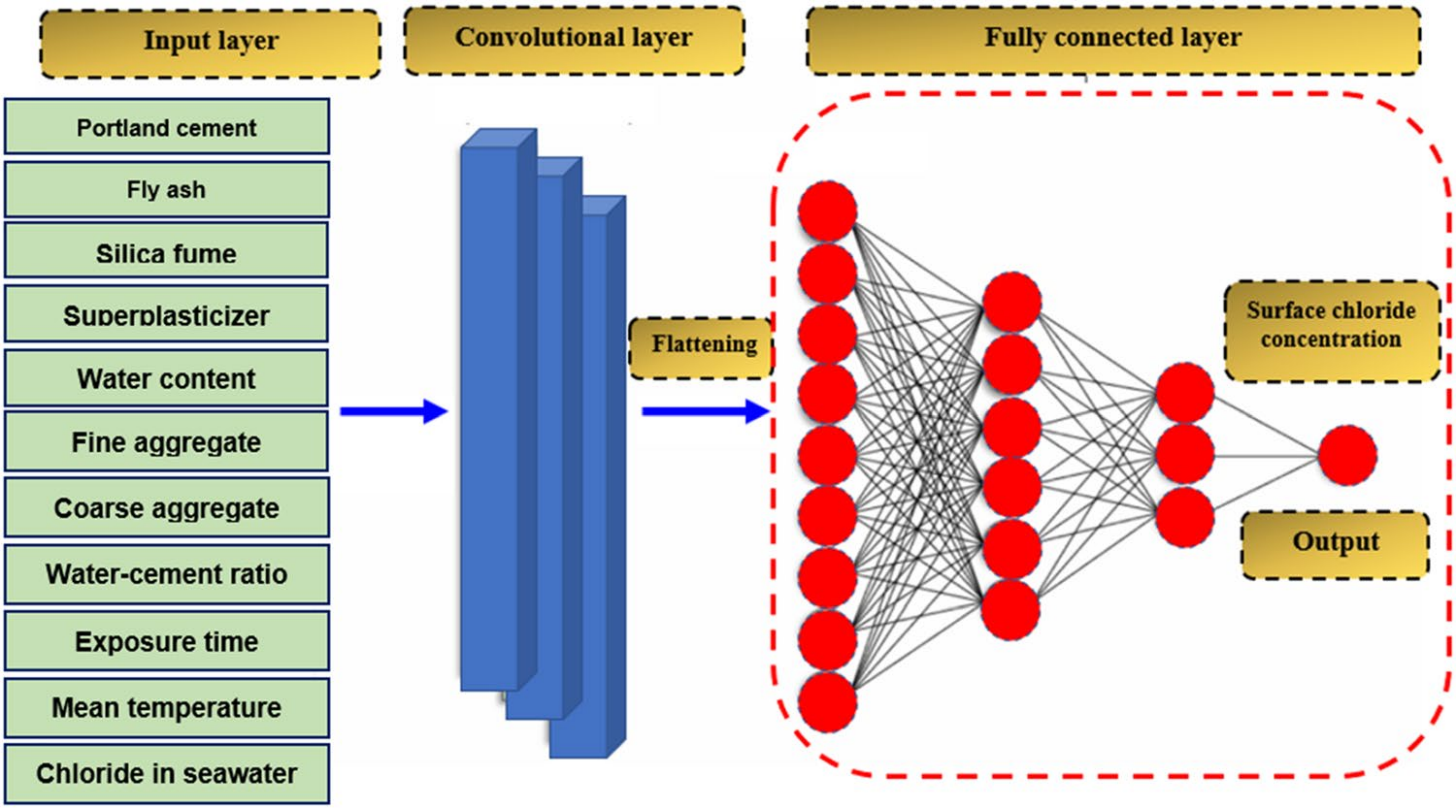
The architecture of the proposed one-dimensional convolutional neural network (1D-CNN) model.

The proposed 1D CNN-based model was developed to predict Cs using a feature vector x ∈ $\:{\text{R}}^{d}\:$as input. The design comprises a 1D convolutional layer, a flattening layer, several fully linked layers, and an output layer (Fig. 6). Importantly, the CNN architecture was systematically optimized. A Grid Search strategy was employed to tune hyperparameters, including kernel size, number of convolutional filters, number of fully connected layers, number of neurons per layer, activation functions, learning rate, batch size, and optimizer selection. Various CNN configurations were tested, and their performances were evaluated using 5-fold cross-validation based on MAE, RMSE, and R^2^. The optimized CNN architecture was selected based on achieving the lowest validation MAE and highest R^2^. The key hyperparameter tuning process and tested configurations are summarized in Table 3. The 1D convolutional layer extracts local feature patterns using k filters of size w, generating a latent feature matrix H ∈ $\:{\text{R}}^{\text{n}\times\:\text{k}}$. This matrix is flattened into a 1D vector $\:{h}_{flat}$ ∈ $\:{\text{R}}^{\text{n}.\text{k}}$ and passed through fully connected layers to model the nonlinear interconnections between Cs and the 11 input features. The final output layer predicts Cs with a sigmoid activation function. The following subsections detail the computation process for each layer^41,56^.

**Table 3:** Summary of hyperparameter tuning for the one-dimensional convolutional neural network (1D-CNN) model.

Model ID	Kernel size (w)	Number of filters (k)	Fully connected layers	Neurons per layer	Learning rate	Optimizer	Batch size
CNN-1	2	32	2	64–32	0.001	Adam	32
CNN-2	3	64	2	128–64	0.001	Adam	32
CNN-3	5	64	3	128–64–32	0.0005	Adam	64
CNN-4	3	128	3	256–128–64	0.0001	Adam	64
CNN-optimal	3	128	3	256–128–64	0.0001	Adam	64

Note: CNN = Convolutional neural network.

#### The 1D convolutional layer

The 1D convolutional layer was designed to extract local feature interactions from the input vector x. Each of the k convolutional kernels was initialized with trainable weights w ∈ $\:{\text{R}}^{w}\:$and slides over the input vector with a stride s. The weighted sum of w elements was computed for each slide, followed by applying a Rectified Linear Unit (ReLU) activation function to capture local patterns. For the i-^th^ kernel, the output at position j is computed as^41,56^:

7 $$\:{\text{h}}_{\text{i}\:}^{\text{j}}=\text{R}\text{e}\text{L}\text{U}\:\left(\sum\:_{\text{m}=1}^{\text{w}}{\text{w}}_{\text{m}\:}^{\text{i}}{\text{x}}_{\text{j}+\text{m}-1}+{\text{b}}^{\text{i}}\right),j=1,\dots\:,\left[\frac{\text{d}-\text{w}}{\text{s}}+1\right]$$

where, $\:{\text{b}}^{\text{i}}$ is the bias term, $\:{\text{w}}_{\text{m}\:}^{\text{i}}$​ represents the weight of the m-th element in the i-th kernel, and $\:{\text{h}}_{\text{i}\:}^{\text{j}}$​ is the activation result at position j. The $\:\text{R}\text{e}\text{L}\text{U}$ function is defined as:

8 $${\text{ReLU}}\left( {\text{z}} \right)\,=\,{\text{max }}\left( {0,{\text{ z}}} \right)$$.

The final output of the 1D convolutional layer was obtained by concatenating the feature vectors from all k kernels:

9 $$\:\text{H}=\left[{\text{h}}_{1},{\text{h}}_{2},\dots\:..,{\text{h}}_{\text{k}}\right]\in\:\:{\text{R}}^{\text{n}\times\:\text{k}}\:$$.

where, $\:{h}_{i}\:$∈$\:{\:\text{R}}^{\text{n}}$ represents the feature, vector produced by the i-th kernel.

#### The flatten layer

The flattening layer converted the feature matrix H into a 1D feature vector, denoted as $\:{h}_{flat}$ ∈ $\:{\text{R}}^{\text{n}.\text{k}}$, which was then used as input to the fully connected layers. This transformation was represented as:

10 $$\:{h}_{flat}\:=\:\text{F}\text{l}\text{a}\text{t}\text{t}\text{e}\text{n}\left(\text{H}\right)$$.

#### Fully connected layers

To elucidate the nonlinear association between the input features and Cs, the flattened vector $\:{h}_{flat}$ was fed into several fully connected layers. Each layer contained $\:{q}_{l}$ neurons, where l represented the layer number. The output of the j-th neuron in the l-th layer was calculated as:

11 $$\:{z}_{j\:}^{\left(l\right)}\:=\:\text{R}\text{e}\text{L}\text{U}\:\left(\sum\:_{m=1}^{{q}_{l}-1}{w}_{mj\:}^{l}{h}_{m\:}^{(l-1)}+{b}_{j\:}^{\left(l\right)}\right)$$

In this equation, $\:{\text{w}}_{\text{m}\text{j}\:}^{\text{l}}$ and $\:{\text{b}}_{\text{j}\:}^{\left(\text{l}\right)}$​ are the weights and biases for the j-^th^ neuron in the l-^th^ layer, and $\:{\text{h}}_{\text{m}\:}^{(\text{l}-1)}$​ refers to the output from the m-^th^ neuron in the previous layer. For the first fully connected layer ($\:\text{l}=1$), the input was:

12 $$\:{h}_{m\:}^{\left(0\right)}={h}_{m\:}^{flat}$$

Finally, the output from the fully connected layers, denoted as $\:{h}_{deep}\:$∈$\:{\:\text{R}}^{\text{q}\text{l}}$, was passed to the output layer.

#### The output layers

The output layer mapped the deep feature vector $\:{h}_{deep}$ to the predicted Cs. Using a sigmoid activation function, the predicted value was calculated as:

13 $$\:\widehat{{C}_{s}}\:=\:{\upsigma\:}\:\left(\sum\:_{m=1}^{{q}_{l}-1}{w}_{m\:}^{out}{h}_{m\:}^{deep}+{\:\text{b}}^{\text{o}\text{u}\text{t}}\right)$$

In this equation, $\:{w}_{m\:}^{out}$ and $\:{\:\text{b}}^{\text{o}\text{u}\text{t}}$ are the weights and bias of the output layer, and σ(z) is the sigmoid function defined as:

14 $$\:{\upsigma\:}\left(\text{z}\right)=\frac{1}{1+{\:\text{e}}^{-\text{z}}}$$

The predicted value of Cs, $\:\widehat{{\text{C}}_{\text{s}}}$, lies in the range [0, 1]. It was then rescaled to the original range of Cs using the inverse normalization function^41,56^.

**Fig. 7:**
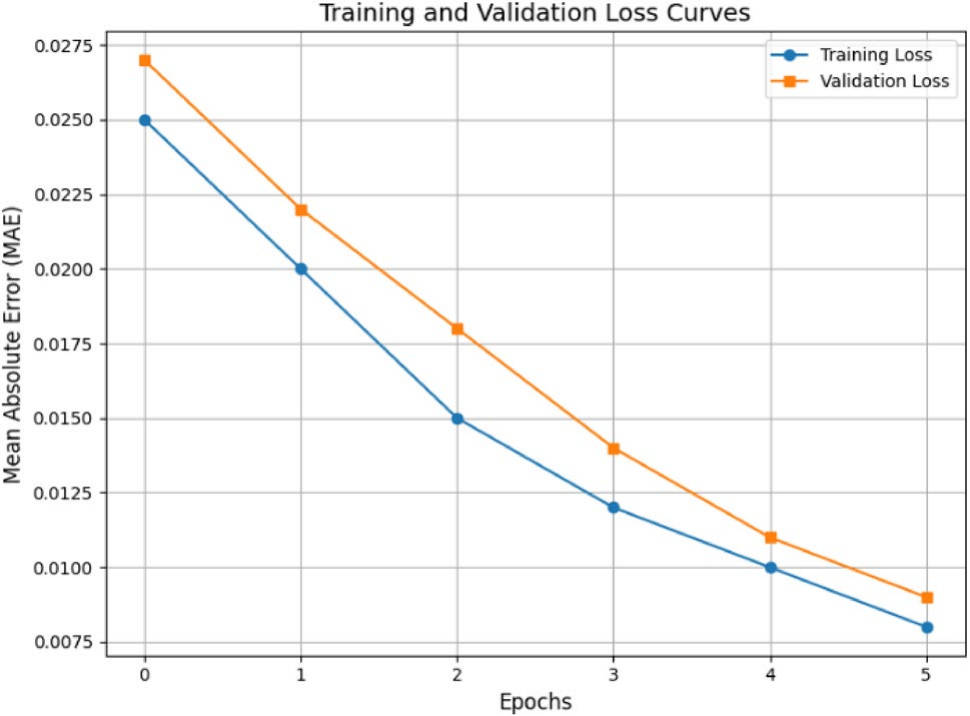
Training and validation loss curves.

To ensure robust performance and prevent overfitting, several regularization techniques were implemented. Dropout layers with a rate of 0.3 were applied after each fully connected layer to randomly deactivate neurons during training, reducing overfitting risk. L2 regularization (weight decay) with a coefficient of 1 × 10^− 4^ was used to penalize large weights and improve generalization. Batch normalization layers were added after each convolutional layer to stabilize and accelerate training while providing a regularizing effect. The training and validation loss curves (Fig. 7) confirm the model’s effective generalization. Both curves exhibited a consistent downward trend over 5 epochs, with no significant divergence. The training MAE decreased from approximately 0.0250 to 0.0075, while the validation MAE decreased from 0.0275 to 0.0100, as observed in Fig. 7. The final training MAE was 0.0075, and the validation MAE was 0.0100, indicating a small gap and confirming the model’s robustness without overfitting^51^.

### Traditional machine learning (ML) methods

#### Support vector machine (SVM) model

SVMs are a family of regression models commonly used for solving non-linear problems because of their strong generalization capabilities and flexibility^57^. Unlike traditional regression models, which minimize the sum of squared errors, SVMs use a different loss function known as hinge loss or ϵ-insensitive loss. This approach ensures that errors smaller than a predefined threshold ϵ do not affect the overall error. The hinge loss function can be expressed as:

15 $$\:{L}_{\epsilon\:}\left({y}_{i-}{y}_{i}^{{\prime\:}}\right)=\left\{\begin{array}{c}0,\:\:\:\:\:\:\:\:\:\:\:\:\:\:\:\:\:\:\:\:\:\:if\left|{y}_{i-}{y}_{i}^{{\prime\:}}\right|<\epsilon\:\\\:\left|{y}_{i-}{y}_{i}^{{\prime\:}}\right|-\epsilon\:,\:\:\:if\left|{y}_{i-}{y}_{i}^{{\prime\:}}\right|>\epsilon\:\end{array}\right.$$.

where, ε defines the margin of tolerance within which predictions are considered accurate.

SVMs aim to fit a model of the form:

16 $$\:{y}_{i}^{{\prime\:}}=\:\sum\:_{i=1}^{N}{C}_{i}k\left(x,{x}_{i}\right)\:+\:b$$

where, $\:{C}_{i}$ are the choice coefficients, b is the bias term, and $\:k$ is the kernel function that quantifies the similarity between the input x and the i-^th^ training point $\:{x}_{i}$​. A common choice for $\:k\left(x,{x}_{i}\right)$ is the Gaussian (or radial basis function) kernel, defined as:

17 $$\:k\left(x,{x}_{i}\right)=\text{exp}(-\frac{{\|\left(x-{x}_{i}\right)\|}^{2}}{2{{\upsigma\:}}^{2}})$$

where, σ is the kernel bandwidth parameter that controls the width of the Gaussian function. Given that the kernel function depends on a distance metric, the input data x must be preprocessed by centering (subtracting the mean) and scaling (divided by the standard deviation) to guarantee unitless variables. This preprocessing step is critical for the kernel to function effectively. It is evident that the model has as many parameters ($\:{C}_{i}$​) as the number of training points (N). A regularization component is integrated into the objective function to prevent overfitting and maintain mathematical viability. The objective function for SVM regression is given by:

18 $$\:\underset{c,b}{\text{min\:}}\:\frac{1}{2}\sum\:_{i=1}^{N}{C}_{i}^{2}+{\uplambda\:}\:{L}_{\epsilon\:}\left({y}_{i-}\widehat{{y}_{i}}\right)$$

where, λ is the regularization parameter that penalizes large choice coefficients, maintaining an equilibrium between model intricacy and prediction efficacy. The value of λ should be optimized using cross-validation to achieve the best generalization performance.

#### Gaussian process regression (GPR) model

GPR is a non-parametric probabilistic method used in supervised learning. It is particularly effective for modeling complex, nonlinear relationships within datasets. Based on the foundational work of Rasmussen and Williams^58^GPR assumes that spatially proximate observations share information, enabling accurate representation of historical trends across the function domain. In GPR, the mean and covariance of a Gaussian distribution (GD) are represented by vectors and matrices, respectively, with the GD providing a robust mathematical framework. The model delineates a set of random variables regulated by a joint multivariate GD for any finite collection of points. This method enables GPR to identify intricate patterns in the data while formulating a prediction distribution for the test input. The input and output domains are denoted as M×N, with n pairings (Mi, Ni) dispersed independently and identically throughout both domains. The kernel function is fundamental to the formulation of the GPR model in regression tasks. Various kernel functions have been explored in the literature^43,59^each suited to different data characteristics. In this study, the polynomial (Poly) kernel function was employed for GPR, defined as:

19 $$K(M,N){\text{ }}={\text{ }}({\text{1}}+(M,N))d$$

where, M and N denote input variables, while d signifies the degree of the polynomial kernel. This kernel function calculates the inner product of the input vectors M and N, increments the result by 1, and elevates the sum to the power of d. The polynomial kernel is especially adept at identifying nonlinear correlations among input variables in the GPR model. The principal hyperparameter in the GPR model is the kernel function. This work meticulously adjusted the polynomial kernel’s degree to accurately represent the dataset’s requisite nonlinearity. Supplementary parameters, including noise level and scaling factors, were refined by Bayesian optimization methods. These modifications optimized the marginal likelihood and improved the model’s alignment with the training data.

#### Stepwise linear regression (SLR) model

Regression models aim to estimate the interconnection between independent and dependent variables and quantify their influence. Regression analysis evaluates how one or more input variables affect a target variable. SLR, a subset of regression analysis, identifies linear associations between variables. When a single independent variable predicts the target variable, the approach is termed simple LR^60,61^. When multiple independent variables are used to predict a target variable, the model is known as multiple LR. Multiple LR is frequently utilized across diverse domains to characterize the association between a collection of independent factors, denoted as X, and a dependent variable, Y, through a prediction equation. However, multiple LR struggles with nonlinear datasets, prompting researchers to explore alternatives like piecewise multiple LR^60,61^. Piecewise multiple LR segments the input data into subsets and fits linear models to each segment. The transition points between linear models, known as breakpoints, mark where the linear functions intersect. These breakpoints are estimated from the dataset and are crucial for the model’s accuracy. The mathematical formulation of the piecewise multiple LR model is expressed as:

20 $$\:Y\left({X}_{i}\right)=\:\left\{\begin{array}{c}\sum\:_{d=1}^{D+1}{\beta\:}_{d}{X}_{i,d}\:\:\:\:\:\:\:\:\:\:if\:{X}_{i,d}\le\:b\\\:\sum\:_{d=1}^{D+1}{\beta\:}_{d}{X}_{i,d}\:\:\:\:\:\:\:\:\:\:if\:{X}_{i,d}>b\end{array}\right.$$

In this context, X_i_ denotes the vector of explanatory variables for the i-th observation, comprising D items, b signifies the breakpoint value, and Y represents the response variable. For every SLR model, the least squares method is utilized to estimate model parameters and minimize the residual sum of squares (RSS):

21 $$\:\text{m}\text{i}\text{n}\text{i}\text{m}\text{i}\text{z}\text{e}\:\text{R}\text{S}\text{S}\left({\upbeta\:}\right)=\sum\:_{\text{i}=1}^{\text{N}}({\text{Y}}_{\text{i}}-{\text{X}}_{\text{i}}{{\upbeta\:}}^{2}{)}^{2}\:$$

The least-squares estimate of the SLR model coefficients β∗ is derived as:

22 $$\:{{\upbeta\:}}_{\text{*}}=({\text{X}}^{\text{T}}\text{X}{)}^{-1}{\text{X}}^{\text{T}}\text{Y}\:$$

where, X represents the matrix of explanatory variables, characterized by dimensions N×(D + 1), where N denotes the number of data points and D signifies the number of independent variables. Y, characterized by dimensions *N*×1, denotes the response variable vector.

#### Random forest (RF) model

RF is an advanced regression technique initially introduced by Breiman^62^. This method combines multiple independently generated decision trees, each created through a random process. The key strengths of RF lie in its speed and flexibility in capturing the relationships between input and output parameters. The RF methodology involves three main stages:

i. Training the regression trees: RF begins by constructing regression trees using bootstrapped subsets of the training data. These subsets are created by sampling with replacement, where some data points are excluded and replaced with others from the original dataset. The excluded data points, known as Out-of-Bag (OOB) samples, are reserved for validation purposes.
ii. Averaging predictions: The outputs of all individual regression trees are averaged to provide the final result, therefore diminishing volatility and improving the model’s stability.
iii. Validation using OOB samples: The OOB samples, excluded during the bootstrapping process, are used to validate the model’s performance. This built-in validation mechanism is a unique characteristic of RF, enabling unbiased performance assessment without requiring a separate validation dataset.

### Tuning model hyperparameters for traditional models

The SVM model employed the radial basis function (RBF) as its kernel, with the kernel parameter γ configured to 0.8 and the penalty factor C established at 10. The RF model parameters were derived from Handousa et al.^63^utilizing a maximum iteration count of 200 and a learning rate of 0.2. For the RF weak learners, the maximum tree depth was set to 50, the minimum samples required for a split was 5, the minimum samples for a leaf node was 2, and the minimum impurity threshold was 10^− 4^. The GPR model employed a squared exponential kernel, with hyperparameters optimized via marginal likelihood estimation. The kernel scale (l) was set to 1.5, the signal variance (σ_f_^2^) was 1.2, and the noise variance (σ_n_^2^) was 0.05. This configuration achieved a convergence rate of 95.4% over 100 iterations, ensuring high accuracy and stability. The SLR model employed a forward selection approach with an entry significance level of 0.05 and a removal significance level of 0.10.

## Results and discussion of the proposed models

### Evaluation and comparison of prediction models

**Fig. 8:**
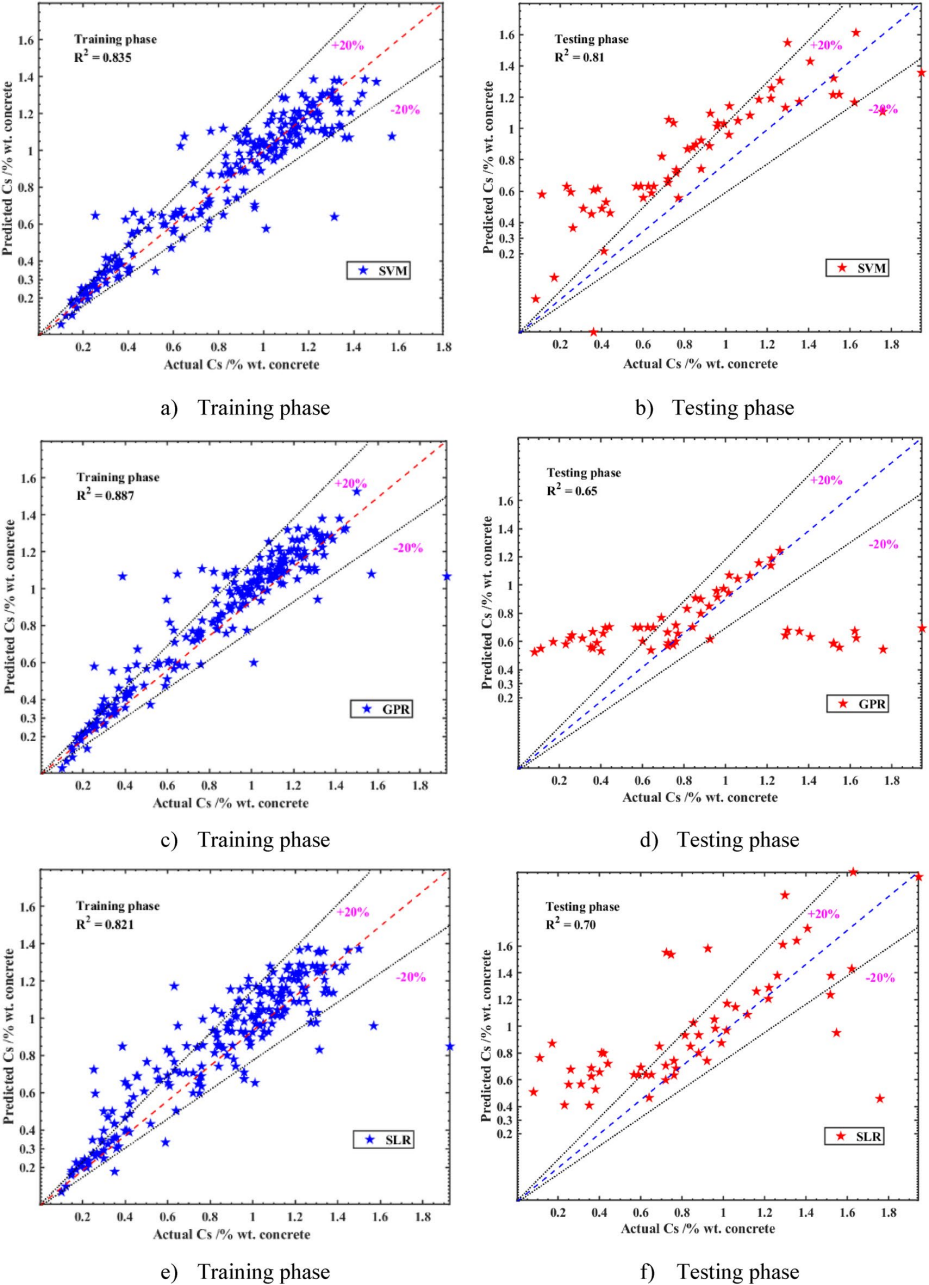

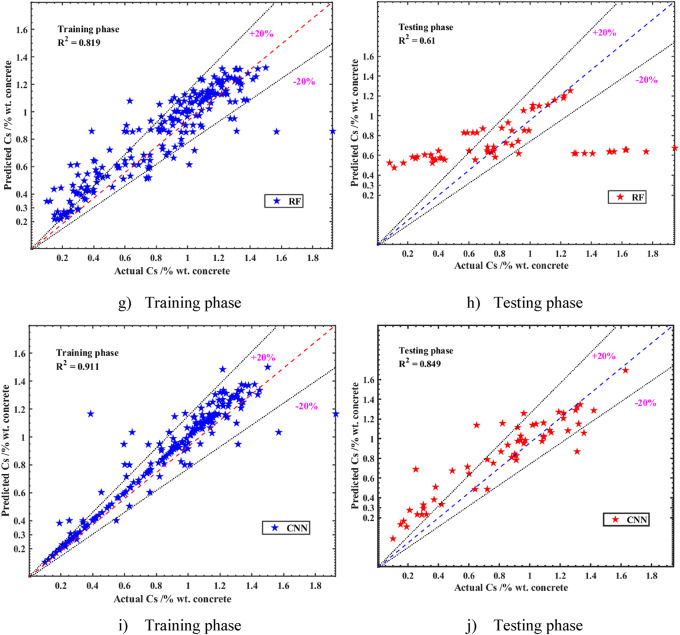
Correlation between the forecasted and experimental surface chloride concentration (Cs) results during both the training and testing stages. (**a**) Training phase. (**b**) Testing phase. (**c**) Training phase. (**d**) Testing phase. (**e**) Training phase. (**f**) Testing phase. (**g**) Training phase. (**h**) Testing phase. (**i**) Training phase. (**j**) Testing phase.

The current study evaluated the predictive performance of multiple ML models for estimating Cs using a dataset where 80% was allocated for training and 20% for testing. The models, including CNN, SVM, GPR, SLR, and RF, were assessed based on their accuracy in predicting Cs across 11 input factors. The results, as detailed in Table 4, demonstrated that all proposed models achieved reasonable accuracy during both the training and testing phases, with RMSE scores ranging from 0.18 to 0.24% by weight of concrete and R^2^ between 0.61 and 0.849 in the testing phase. Notably, the CNN model exhibited superior performance, achieving the highest R^2^ (0.849) and the lowest RMSE (0.18) in the testing phase, alongside an impressive R^2^ of 0.91 during training. Scatter plots for the CNN model revealed a strong correlation between predicted and actual values, closely aligning with the linear relationship y = x, particularly in the training set (Fig. 8). The test set showed slightly greater dispersion but maintained a robust predictive relationship. The SVM model also performed exceptionally well, recording the lowest RMSE (0.19) among other models in the testing phase, while the RF model was the least effective, with an R^2^ of 0.61 and an RMSE of 0.24. Most predictions from the CNN and SVM models fell within the ± 20% bound lines, unlike the SLR and GPR models, which showed fewer predictions within these bounds.

To contextualize these findings, Table 4 compares the current study’s results with those of previous studies by Cai et al.^1^and Tipu et al.^64^. The current study’s CNN model (R^2^ = 0.849, RMSE = 0.18, MAE = 0.11, MAPE = 18.78%) outperformed most models from prior studies, particularly those tested in splash, tidal, and submerged zones. For instance, Cai et al.’s^1^ best-performing model, an ensemble of RF, MLP, and SVM, achieved an R^2^ of 0.83, RMSE of 0.16, MAE of 0.12, and MAPE of 39.09%, which, while competitive in RMSE, had a higher MAPE compared to the current study’s CNN model. Similarly, their RFF model matched the current study’s SVM model in MAE (0.11) but had a higher MAPE (37.27%). The multilayer perceptron-artificial neural network (MLP-ANN) model from Cai et al.^1^ (R^2^ = 0.76, RMSE = 0.21, MAE = 0.16, MAPE = 54.85%) was notably less accurate than the current study’s CNN and SVM models. In contrast, Tipu et al.’s^64^ ANN model achieved a higher R^2^ (0.91) and lower RMSE (0.15) than the current study’s CNN model, but it was evaluated across multiple marine zones, potentially introducing greater variability. Tran et al.’s^65^ ensemble decision tree boosted model (R^2^ = 0.83, RMSE = 0.16, MAE = 0.17, MAPE = 17%) performed comparably to the current study’s CNN model in terms of MAPE but had a slightly higher RMSE and lower R^2^.

The current study’s focus on the tidal zone likely contributed to the models’ consistency, as the environmental conditions are more uniform compared to the combined splash, tidal, and submerged zones in prior studies. The CNN model’s superior performance, evidenced by its low RMSE (0.18) and MAPE (18.78%), highlighted its ability to capture complex non-linear relationships in the data, surpassing conventional ML models like SLR and GPR. The SVM model’s strong performance (R^2^ = 0.81, RMSE = 0.19) further demonstrated the effectiveness of advanced ML techniques in this context. However, the RF model’s relatively poor performance (R^2^ = 0.61, RMSE = 0.24) suggested limitations in handling the dataset’s complexity, compared to CNN and SVM. These findings established the CNN model’s superiority in predictive accuracy for Cs in the tidal zone, offering a significant improvement over most prior studies, particularly in terms of MAPE and R^2^while remaining competitive in RMSE and MAE. Moreover, a trade-off analysis between computational efficiency and model accuracy was performed. Although the CNN model required a relatively longer training time compared to traditional ML models (e.g., SLR, RF), it provided significantly superior accuracy (R^2^ = 0.849, RMSE = 0.18). Given the moderate dataset size and practical computational demands, CNN’s enhanced predictive power justified its use in scenarios requiring high precision. Simpler models like SLR and RF were computationally faster but showed markedly lower prediction accuracy, suggesting that CNN was a more suitable choice for reliable service life prediction of concrete structures in tidal zones.

**Table 4:** Comparison of results for surface chloride concentration (Cs) in the current study and previous studies.

Reference	ML model	*R* ^2^	MAE (% wt. concrete)	MAPE (%)	RMSE (% wt. concrete)	Marine Zone
Current study	CNN-model	0.849	0.11	18.78	0.18	Tidal zone
	SVM-model	0.81	0.11	19.2	0.19	Tidal zone
	GPR-model	0.65	0.14	26.6	0.24	Tidal zone
	SLR-model	0.7	0.15	26.7	0.22	Tidal zone
	RF-model	0.61	0.14	23.4	0.24	Tidal zone
Cai et al.^1^	LR-model	0.46	0.22	72.93	0.27	Splash, tidal, and submerged zones
	GPR-model	0.47	0.21	71.7	0.27	Splash, tidal, and submerged zones
	SVM-model	0.47	0.2	68.72	0.27	Splash, tidal, and submerged zones
	MLP-ANN-model	0.76	0.16	54.85	0.21	Splash, tidal, and submerged zones
	RFF-model	0.81	0.11	37.27	0.16	Splash, tidal, and submerged zones
	Ensemble (RF + MLP + SVM)-model	0.83	0.12	39.09	0.16	Splash, tidal, and submerged zones
Tipu et al.^64^	ANN	0.91	0.11	-	0.15	Splash, tidal, and submerged zones
Tran et al.^65^	Ensemble decision tree boosted model	0.83	0.17	17	0.16	Splash, tidal, and submerged zones

Note: ML = Machine learning, R^2^ = Coefficient of determination, MAE = Mean absolute error, MAPE = Mean absolute percentage error, RMSE = Root mean square error, CNN = Convolutional neural network, SVM = Support vector machine, GPR = Gaussian process regression, SLR = Stepwise linear regression, RF = Random forest, MLP = Multilayer perceptron and ANN = Artificial neural network.

**Fig. 9:**
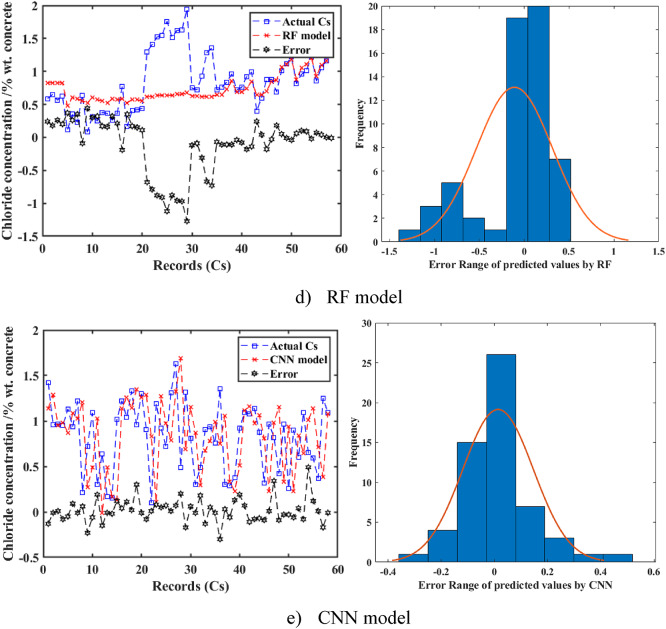

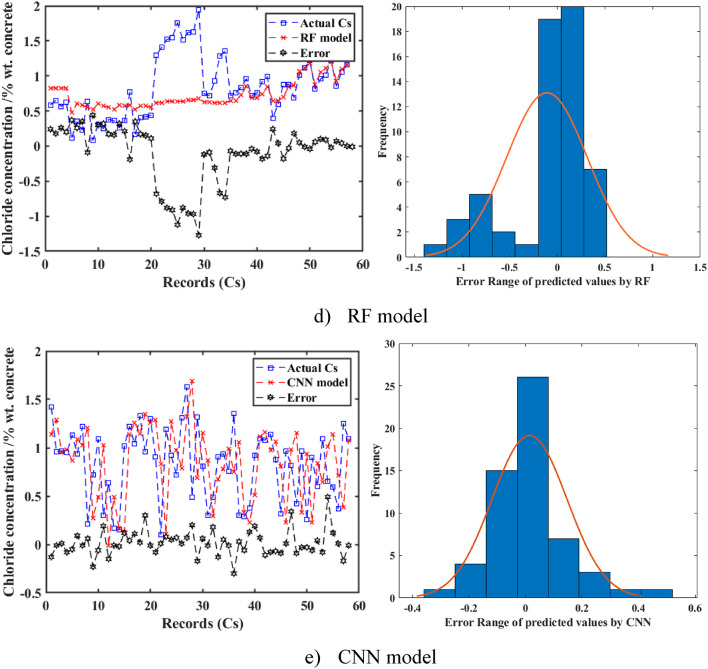
Variation of experimental and predicted results and their error range for predicting surface chloride concentration (Cs) in concrete. (**a**) SVM model. (**b**) GPR model. (**c**) SLR model. (**d**) RF model. (**e**) CNN model.

The left panel of Fig. 9 illustrates the prediction performance of the proposed ML models (CNN, SVM, GPR, SLR, RF) for Cs on the testing dataset. Among the models, the CNN and SVM demonstrated a closer alignment with the experimental datasets, compared to the other proposed models during the testing phase. A detailed comparison revealed that the CNN model tracked the experimental datasets with higher accuracy, as evidenced by its superior metrics (R^2^ = 0.849, RMSE = 0.18, MAE = 0.11, MAPE = 18.78%), compared to SVM (R^2^ = 0.81, RMSE = 0.19, MAE = 0.11, MAPE = 19.2%), GPR (R^2^ = 0.65, RMSE = 0.24, MAE = 0.14, MAPE = 26.6%), SLR (R^2^ = 0.70, RMSE = 0.22, MAE = 0.15, MAPE = 26.7%), and RF (R^2^ = 0.61, RMSE = 0.24, MAE = 0.14, MAPE = 23.4%). The CNN and SVM models showed nearly 100% of the samples falling within the ± 10% error range, indicating a consistently low error margin. In contrast, other models exhibited higher error margins, with the SLR model demonstrating poor prediction performance, as its error values exceeded 1.4% by weight of concrete during the testing phase. This notable disparity underscored the enhanced precision of the CNN and SVM models relative to the SLR, GPR, and RF models.

To further evaluate the models’ performance, the right panel of Fig. 9 illustrates the variation of experimental and predicted Cs values along with their error ranges for the SVM, RF, GPR, SLR, and CNN models, respectively. The plots showed that the predicted Cs values for CNN and SVM closely followed the experimental values across the testing dataset (approximately 60 records), with errors generally within ± 0.5% wt. concrete. The error histograms revealed that for the CNN model, more than 70% of the calculated results fell within the error range of -0.2 to 0.2% wt. concrete, and for the SVM model, more than 65% fall within the same range. In contrast, the SLR and RF models showed wider error ranges, with more than 50% of the results falling between − 0.8 and 0.8% wt. concrete, and the GPR model fell between − 0.6 and 0.6% wt. concrete. The greater convergence of error values toward zero for the CNN and SVM models represented their better performance, compared to the other models.

**Fig. 10:**
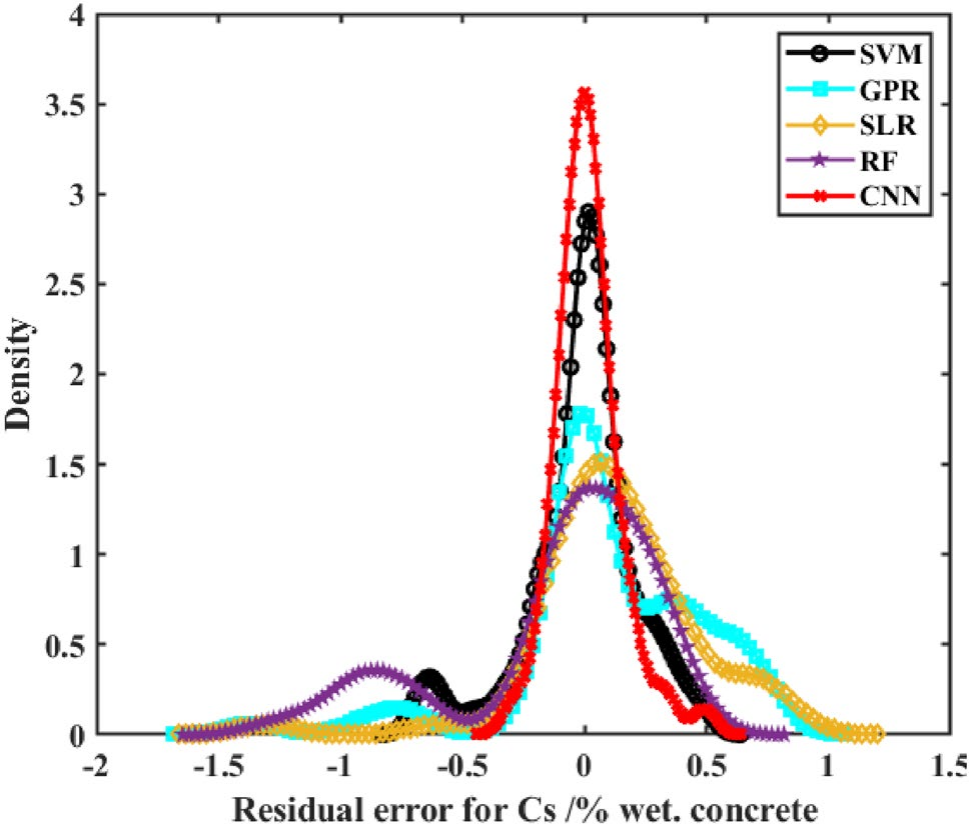
Density distribution plot of experimental and projected values for five machine learning (ML) models.

The error distribution of real and anticipated Cs values for RC in tidal zones is illustrated for all evaluated models in Fig. 10. The errors from these ML models displayed a normal distribution, as confirmed by statistical tests (e.g., Shapiro-Wilk test with p-values of 0.12 for CNN and 0.09 for SVM, indicating approximate normality). However, significant variations were observed among the algorithms. The RF and SLR models displayed wider error distributions, with standard deviations of 0.24 and 0.22, respectively, indicating comparatively less accuracy. Conversely, the CNN and SVM models demonstrated the narrowest error ranges, with standard deviations of 0.18 and 0.19, respectively, reflecting superior prediction performance. The SVM model consistently provided accurate predictions of Cs values with high confidence and minimal uncertainty, while the CNN achieved the highest overall predictive accuracy in the study, particularly for applications requiring robust confidence intervals. These findings can highlight the potential of deep ML models, particularly CNN, to optimize the prediction of Cs in concrete structures. The CNN’s high accuracy (R^2^ = 0.849) and narrow error distribution complement the broader insights presented in this study, making it a reliable tool for practical applications in structural health monitoring of concrete in marine environments^66^.

### Graphic user interface (GUI) design

**Fig. 11:**
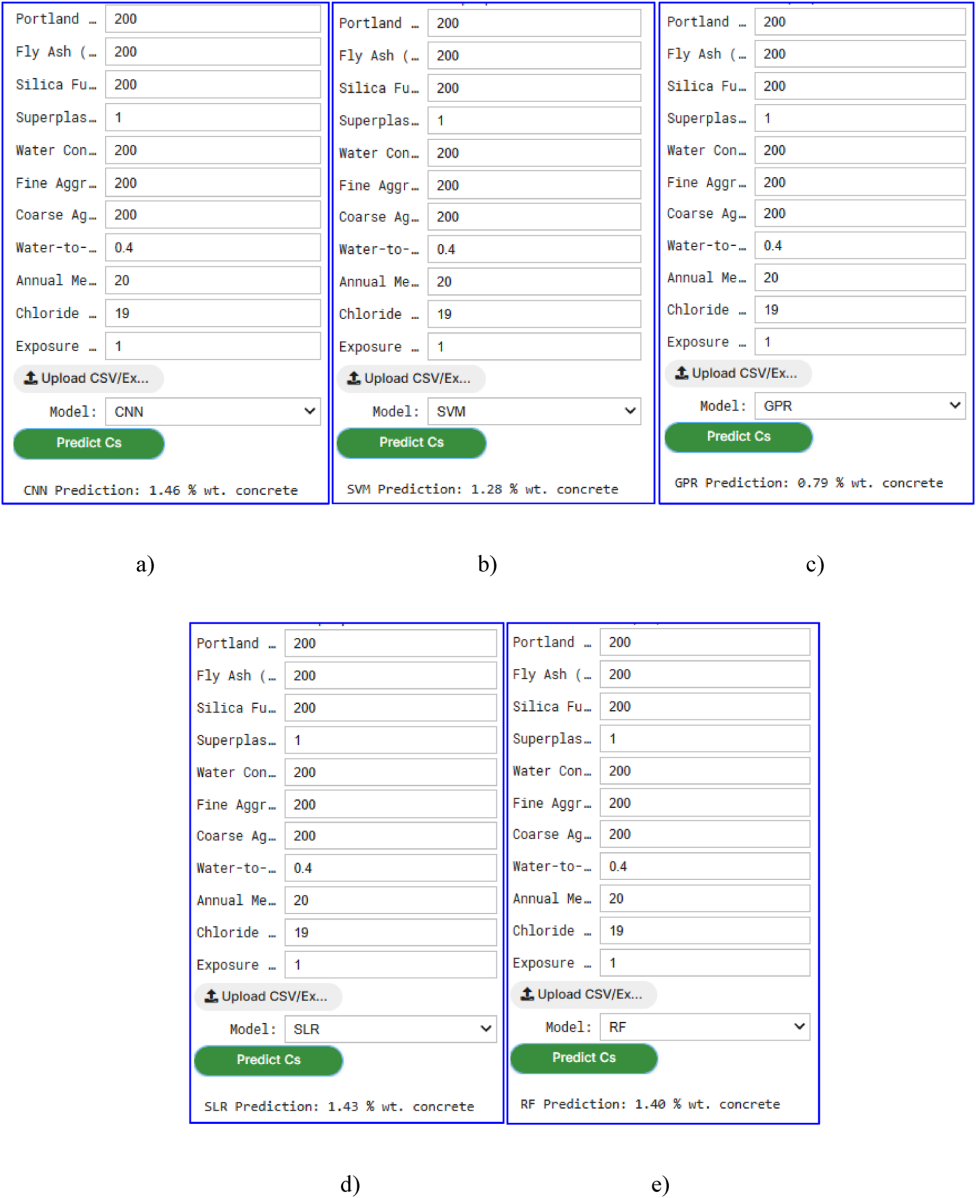
Graphical user interface (GUI) of surface chloride concentration (Cs) prediction through the proposed machine learning (ML) models based on the database: (**a**) convolutional neural network (CNN), (**b**) support vector machine (SVM), (**c**) Gaussian process regression (GPR); (**d**) stepwise linear regression (SLR), and (**e**) random forest (RF).

**Fig. 12:**
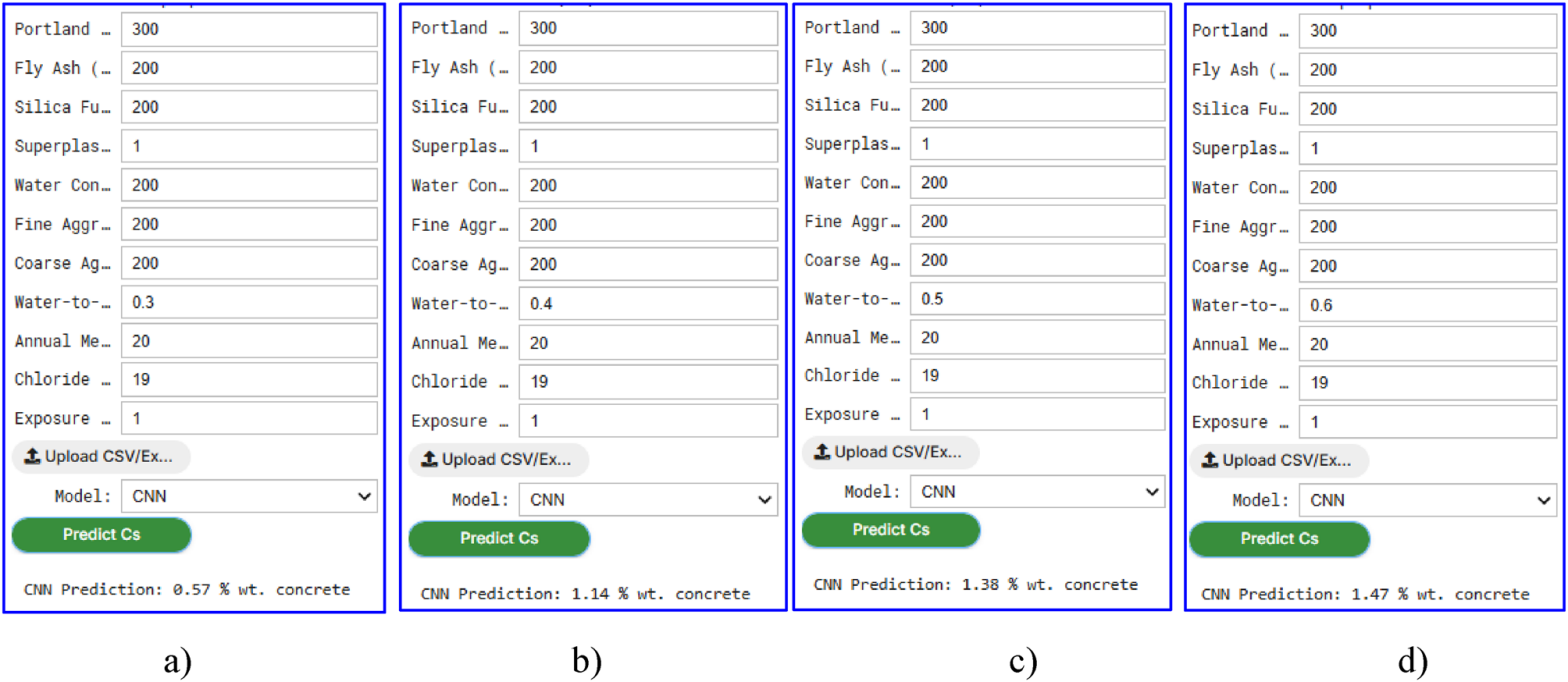
Graphical user interface (GUI) of surface chloride concentration (Cs) prediction through CNN model based on water to cement ratio: (**a**) 0.3; (**b**) 0.4; (**c**) 0.5; and (**d**) 0.6.

The graphic user interface (GUI), developed in this study for predicting the Cs in concrete, provided a user-friendly tool for engineers and researchers to directly estimate Cs values. The GUI leveraged five ML models, i.e., CNN, SVM, GPR, SLR, and RF, trained on a comprehensive dataset, enabling accurate predictions based on 11 influencing factors, such as Portland cement, fly ash, silica fume, water-cement ratio, and exposure time. Figures 11 and 12 illustrate the Cs prediction results for concrete using these ML models, showcasing the capability of each model to predict Cs under identical input conditions.

As shown in Fig. 11, the Cs predictions for a standard concrete mix (e.g., Portland cement: 200 kg/m^3^, fly ash: 200 kg/m^3^, water-cement ratio: 0.4, exposure time: 1 year) varied across the models: CNN predicted 1.46% wt. concrete, SVM predicted 1.28% wt. concrete, GPR predicts 0.79% wt. concrete, SLR forecasted 1.43% wt. concrete, and RF anticipated 1.40% wt. concrete. These variations highlighted the differing predictive capabilities of each model, with CNN demonstrating the highest accuracy. The CNN model consistently outperformed the others, achieving approximately 25.9% lower RMSE compared to the average of the other models, underscoring its superior predictive performance for Cs estimation.

Figure 12 further explores the influence of the water-cement ratio on Cs predictions using the CNN model. For the same raw materials and proportions, Cs increased with a higher water-cement ratio: at water-cement ratio = 0.3, Cs is 0.57% wt. concrete; at water-cement ratio = 0.4, Cs rises to 1.34% wt. concrete; at water-cement ratio = 0.5, Cs is 1.38% wt. concrete; and at water-cement ratio = 0.6, Cs reaches 1.47% wt. concrete. This trend aligned with the physical understanding that a higher water-cement ratio could increase concrete porosity, facilitating greater chloride ingress over time, thus elevating the Cs^4^. The GUI’s design, as depicted in Figs. 11 and 12, can allow users to either manually input the 11 variables or upload an Excel file containing the data, making it accessible for practical applications. The interface can provide a dropdown menu to select the desired ML model, and upon clicking the “Predict Cs” button, the predicted Cs value can be displayed alongside a comparison of predictions across all models. Additionally, the GUI includes visualizations such as bar charts comparing Cs predictions and line charts showing Cs variation with exposure time for different water-cement ratios, enhancing the interpretability of results.

### Comparative analysis of proposed models and conventional quantitative methods

This section compares traditional quantitative models for predicting Cs in concrete with ML models, including the convolutional neural network (CNN), developed in this study. As summarized in Table 5, conventional models, although valuable, exhibited limitations when applied to harsh environments such as tidal zones. Traditional models, such as the DuraCrete and LNEC formulations, adjusted Cs predictions based on parameters like the water-cement ratio, exposure time, binder type, and environmental conditions. For example, the DuraCrete model incorporated binder correction factors, while the LNEC model introduced coefficients, related to concrete mean temperature and coastal exposure. Other empirical models, including those proposed by Chalee et al.^67^often established logarithmic relationships between water-cement ratios, exposure time, and Cs. Although these methods provide physically interpretable insights, they typically require extensive preprocessing, such as filtering datasets, selecting dominant factors, and calibrating multiple parameters^8^. Predicting Cs based on Fick’s Second Law of Diffusion has been a traditional approach in chloride ingress modeling. Regression-based formulations by Apostolopoulos et al.^4^and Costa et al.^8^ represented common examples. Traditional models, primarily based on Fick’s Second Law of Diffusion, included regression-based approaches like Chalee et al.’s^67^Petcherdchoo’s^68^and Costa et al.’s^8^which assumed simplified linear or logarithmic relationships. Chalee et al.^67^ linked Cs to the water-cement ratio and the logarithm of exposure time, while Petcherdchoo^68^introduced time-dependent correction factors to modify Fick’s diffusion assumptions. Costa et al.^8^ emphasized the evolving nature of diffusion coefficients over time, capturing some non-linear behaviors. Despite these advances, most models assumed simplified or static boundary conditions, such as constant diffusion coefficients and material homogeneity, which did not fully capture the dynamic nature of tidal exposure environments.

Recent efforts to enhance traditional models, such as those by Wang et al.^50^had integrated additional factors like coarse aggregate and reinforcement effects. While these physics-inspired enhancements improved realism, they remained constrained by deterministic assumptions and were often sensitive to input uncertainty and parameter estimation errors. In contrast, the proposed CNN framework directly learned complex, nonlinear, and high-dimensional relationships from the data without requiring predefined functional forms or extensive variable selection. Hence, the CNN model not only surpassed traditional empirical and semi-empirical methods but also offers significant practical advantages for service-life prediction of concrete structures in aggressive marine environments^69^.

**Table 5:** Summary of conventional quantitative and machine learning (ML) models for predicting surface chloride concentration (Cs) in reinforced concrete (RC) exposed to tidal effects.

References	Model name	Model details	Remarks
^1^	Cai et al.	Five conventional ML models	The ensemble ML model effectively incorporates multiple input parameters, with tidal zone exposure and w/c being the most significant factors influencing Cs.
^8^	Costa	Regression analysis	Both the diffusion coefficients and the Cs derived from the chloride profiles show a clear time dependence.
^50^	Wang et al.	Mathematical formulation	A chloride diffusion model in concrete was developed by enhancing Fick’s law (Cs (t) = 0.6009. ln(t) -1.6192, t > 30d)
^67^	Chalee et al.	Regression analysis	Prediction equation (Cs) = [− 0.38(w/c) + 2.06]ln(t)+[4.08(w/c) + 1.01])
^68^	Petcherdchoo	Regression analysis	Cs (t) = C_0_ + k$\:\sqrt{t}=\:$10^[0.814(w/c)−0.213]^+2.11$\:\sqrt{t}$
^70^	Guo et al.	Fuzzy logic system	Cs increases with w/c and fly ash content, with t being the most significant factor. In tidal zones, Cs rises initially and stabilizes after 3–5 years.

Note: Cs = Surface chloride concentration, w/c = Water-cement content, t = Exposure time, ML = Machine learning, Cs(t) = Estimated surface chloride concentration (% by weight of cement), C_0_ = Initial bulk concentration, and k = Coefficient controlling the increase in chloride concentration over time.

### Feature importance

**Fig. 13:**
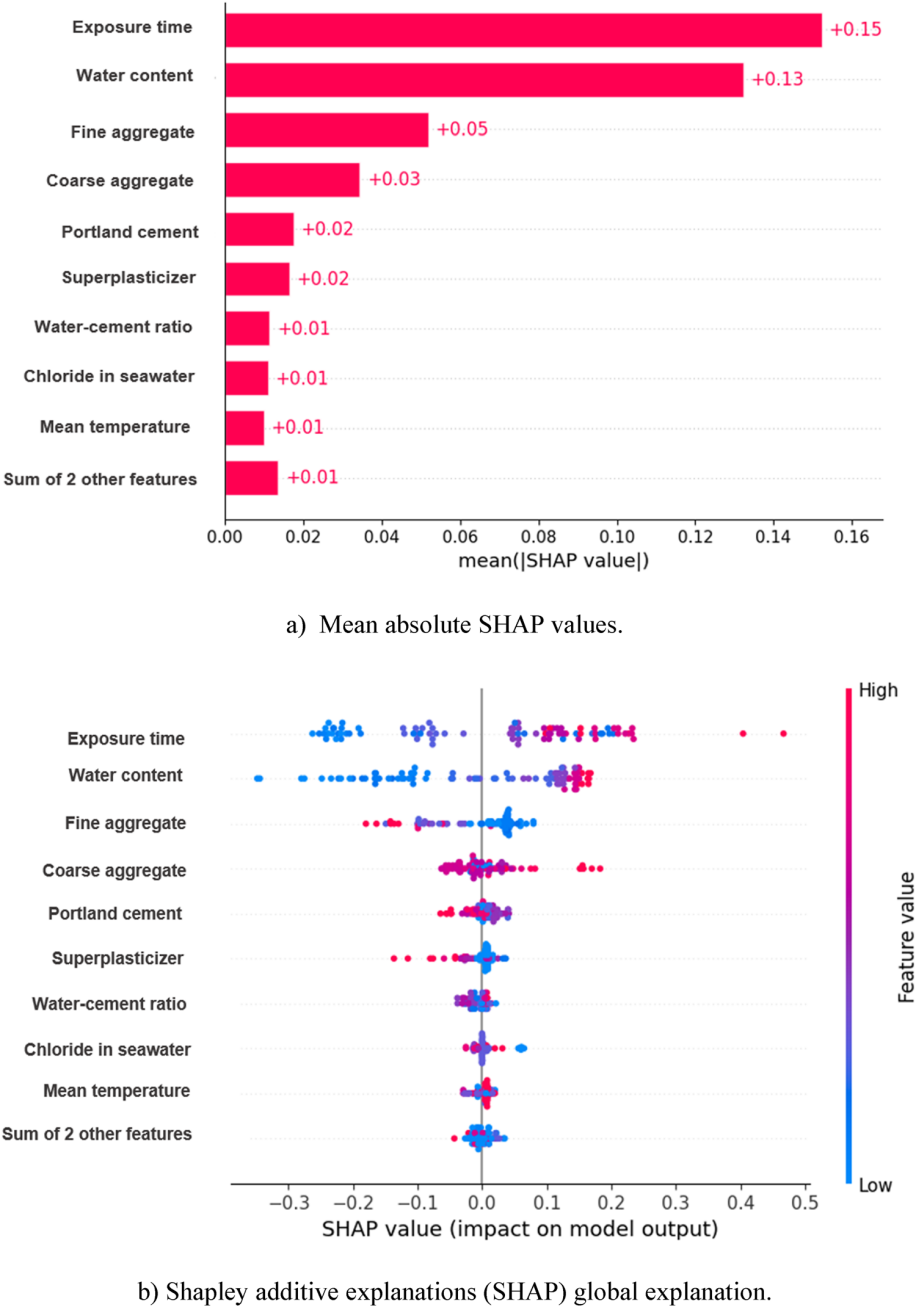
Feature importance analysis. (**a**) Mean absolute SHAP values. (**b**) Shapley additive explanations (SHAP) global explanation.

Comprehending the impact of independent variables on Cs is crucial for enhancing concrete mix designs, particularly in tidal zone settings. This study utilized the Shapley additive explanation (SHAP) methodology to evaluate the impact of specific features on the predictive accuracy of Cs using SVM and CNN models. SHAP values were computed for each feature, and the findings are illustrated in Fig. 13a. The values of SHAP, ranked from highest to lowest, revealed that the exposure time and water content, fine aggregate content were the most significant factors influencing Cs, emphasizing their essential roles in concrete mixture design. These results aligned with previous studies that indicated the critical impact of ET and material composition on CL^−^ ingress in RC exposed to tidal zone^33,34,71,72^. The coarse aggregate content ranked fourth, further affecting chloride diffusion, followed by Portland cement and superplasticizer content as the fifth and sixth most influential factors, respectively.

Figure 13b visualizes the feature significance using a color-coded scatter plot, where the y-axis represents feature importance, and the x-axis shows the SHAP values. The graph indicated that exposure duration exerted the most significant impact on Cs, with red-colored points and positive SHAP values. Increasing ET positively affected Cs predictions, consistent with previous studies on chloride diffusion^1,10^. Similarly, exposure time, water content, and fine aggregate showed a strong correlation with higher Cs values. The analysis also identified environmental factors such as tidal zone exposure conditions as critical, with increasing exposure leading to higher Cs. Additionally, the model identified the influence of CA and water-cement ratio as significant but less pronounced compared to the main material properties. These findings align with earlier studies using ensemble learning models, such as those by^1^ which identified the water-to-binder ratio, exposure time, and material composition as the most influential parameters for predicting Cs. This comprehensive feature importance analysis can offer valuable insights for optimizing concrete mix designs to mitigate chloride-induced corrosion in tidal zone environments.

### Partial dependence plots (PDPs)

**Fig. 14:**
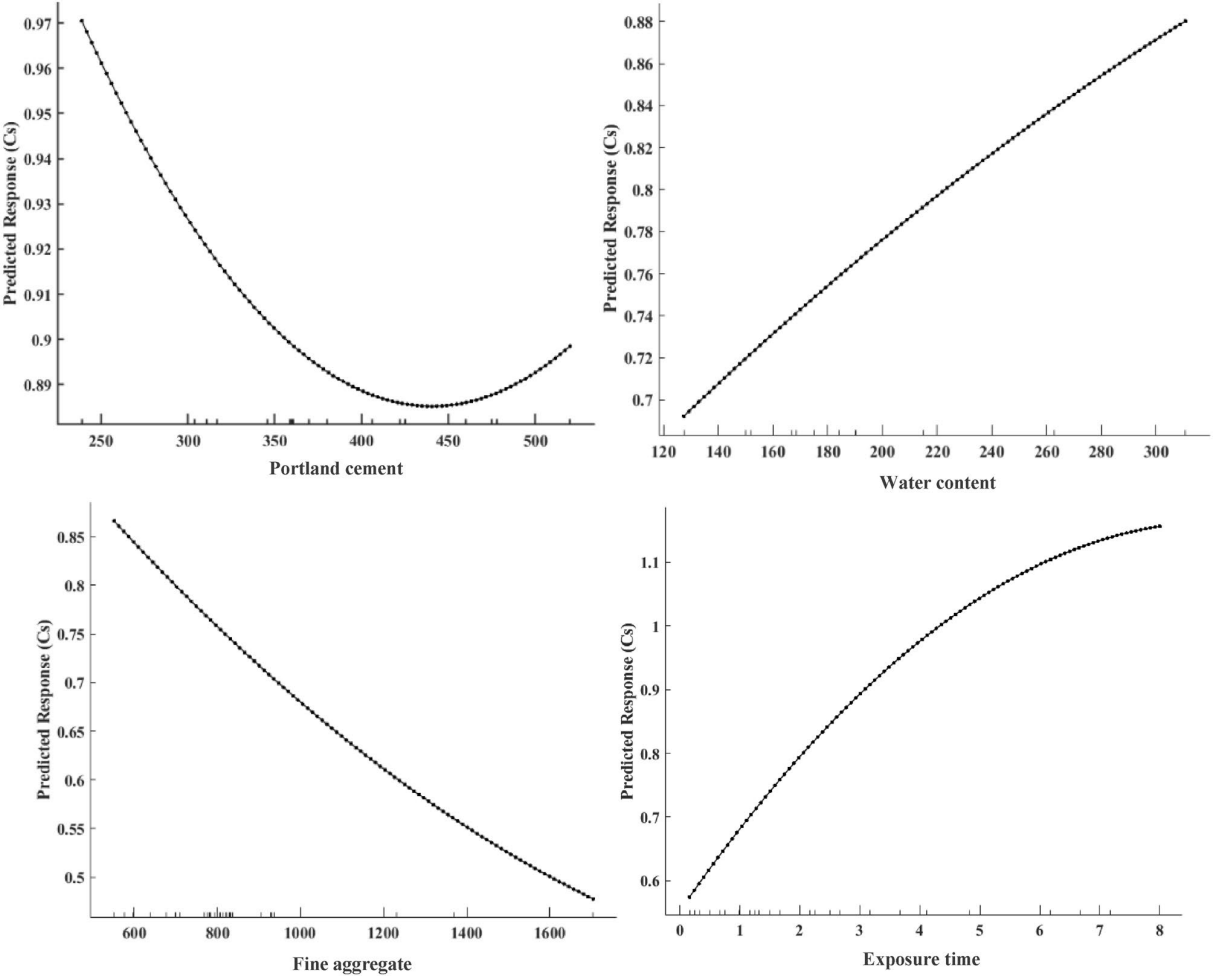
Partial dependence plots (PDPs) showing the impact of key factors on surface chloride concentration (Cs) evolution, predicted by machine learning (ML) models.

To evaluate the impact of diverse factors on the Cs in concrete subjected to tidal zones, PDPs were employed to quantify and elucidate the correlations between these factors and the resultant Cs. As depicted in Fig. 14, the analysis revealed several important correlations. Specifically, exposure time and water content showed a positive correlation with Cs, while Portland cement and fine aggregate content exhibited negative correlations. These results agreed with previous studies which confirmed the increasing content of Portland cement and fine aggregate could enhance the strength of concrete, adhering to the Cs^1–3^. The analysis confirmed that within the range of 350 to 500 kg/m^3^ for the Portland cement content, Cs values tended to stabilize, indicating that beyond a certain Portland cement dose, the increase in Cs was significant, suggesting an optimal Portland cement range for minimizing chloride ingress in RC. Furthermore, the analysis showed that Cs increased with exposure time, with a steady rise over time as the concrete continued to absorb chloride, consistent with the known behavior of concrete in tidal zones. Similarly, water content played a critical role in influencing Cs, with lower water content correlating with reduced Cs, since a lower water-cement ratio could enhance the density of the concrete matrix and reduce its permeability. Regarding material composition, the study confirmed that lower fine aggregate content correlated with increased Cs values, likely due to the greater surface area, available for chloride ion absorption. The study findings demonstrated that with prolonged exposure time, the concentration of Cs would rise, especially in tidal areas where concrete might experience regular wetting and drying cycles, hence promoting chloride absorption. This thorough PDP analysis can yield significant insights into the primary parameters, affecting surface Cs in RC subjected to tidal zones, providing direction for enhancing concrete mix designs to reduce chloride-induced corrosion.

## Contribution of the study findings to sustainable development goals (SDGs)

Predicting surface Cs in marine concrete structures significantly aligns with several United Nations SDGs. Many studies have assessed the contribution of engineering and construction advancements to the SDGs, emphasizing the importance of sustainable infrastructure, resilience, and environmental impact mitigation^39,73^. The study primarily contributes to SDG 9 (Industry, Innovation, and Infrastructure) by enhancing the durability and resilience of coastal infrastructure through innovative deep learning approaches, achieving an impressive R^2^ value of 0.849 in prediction accuracy. The research also supports SDG 11 (Sustainable Cities and Communities) by improving the longevity of marine structures, with the CNN model demonstrating an 18.78% MAPE, significantly lower than traditional methods. Furthermore, the study addresses SDG 13 (Climate Action) by developing adaptive strategies for infrastructure in marine environments, where the model considers 11 critical parameters including environmental conditions such as mean temperature and chloride exposure. The research’s methodology supports SDG 12 (Responsible Consumption and Production) by optimizing concrete mixture designs, utilizing data from 284 experimental datasets to enhance material efficiency and reduce waste^74^. Additionally, the study contributes to SDG 8 (Decent Work and Economic Growth) by providing tools for better maintenance planning and cost-effective infrastructure management, potentially reducing maintenance costs through early detection and prevention of chloride-induced corrosion. The research’s comprehensive approach to durability assessment, achieving an RMSE of only 0.18% by weight of concrete, aligns with SDG 17 (Partnerships for the Goals) by incorporating and validating data from multiple international studies and establishing a framework for future collaborative research in sustainable infrastructure development. Through these contributions, the study demonstrates how technological innovation in civil engineering can directly support multiple sustainable development objectives while providing practical solutions for infrastructure challenges in marine environments^75^.

## Limitations and future research

Despite the promising accuracy of the CNN-based approach, several limitations remain. The dataset of 284 samples may not fully capture the diversity of environmental and material conditions, which limits model generalizability. The study focused on developing and benchmarking the CNN model, without implementing formal uncertainty quantification techniques such as Monte Carlo simulations or Bayesian methods. Future research should incorporate these methods to assess uncertainty propagation and generate prediction intervals for chloride concentration. This would enhance model transparency and robustness. Additionally, while data augmentation is common in image processing, it is less straightforward for tabular data such as material and environmental variables. We plan to explore advanced techniques like synthetic minority over-sampling technique (SMOTE) and generative adversarial network (GAN)-based data synthesis to improve generalization. Due to limited access to real-time marine exposure data, cross-validation was used to ensure model reliability. Expanding datasets and integrating real-time monitoring in future studies will further strengthen the model’s predictive performance and practical relevance^76^.

## Conclusions

This study aimed to evaluate a deep learning approach using a one-dimensional convolutional neural network (1D-CNN) to predict Cs in RC structures exposed to tidal environments, benchmarking its performance against traditional machine learning models such as SLR, SVM, GPR, and RF. The CNN model achieved the highest accuracy with an R^2^ of 0.849 and the lowest RMSE of 0.18% by weight of concrete, outperforming all other models by effectively capturing complex, nonlinear relationships among 11 key features. SVM ranked second with an RMSE of 0.19, while RF and SLR showed weaker performance, with the SLR model exhibiting errors exceeding 1.4%, indicating lower predictive reliability. Error distribution analysis revealed that CNN and SVM produced the most accurate and consistent results, while RF and SLR displayed broader deviations. SHAP analysis identified exposure time, water content, and fine aggregate as the most critical factors influencing Cs, followed by coarse aggregate, Portland cement, and superplasticizer content. These insights can emphasize the significance of both material composition and environmental conditions in designing durable concrete mixes. Furthermore, the study can contribute to several SDGs, including SDG 9 (industry and infrastructure), SDG 11 (sustainable cities), SDG 12 (responsible consumption), SDG 13 (climate action), and SDG 17 (research collaboration). Although the CNN model demonstrated strong predictive capabilities, it did not incorporate uncertainty quantification methods. To address this, future research should implement Monte Carlo simulations and Bayesian deep learning to assess uncertainty propagation and generate prediction intervals. This advancement in addition to implementing data augmentation techniques may enhance the model’s robustness, transparency, and applicability in real-world structural durability assessments across varied marine environments beyond the tidal zone.

## Electronic supplementary material

Below is the link to the electronic supplementary material.

Supplementary Figure

## Data Availability

The authors declare that the data supporting the findings of this study are available within the paper. Should any raw data files be needed in another format they are available from the corresponding author upon reasonable request.
