# Supplementary figures and images for: A convolutional neural network-based deep learning approach for predicting surface chloride concentration of concrete in marine tidal zones
Source: Sci Rep. 2025 Jul 29;15:27611. doi: 10.1038/s41598-025-12035-1 (PMC12307714; doi:10.1038/s41598-025-12035-1)

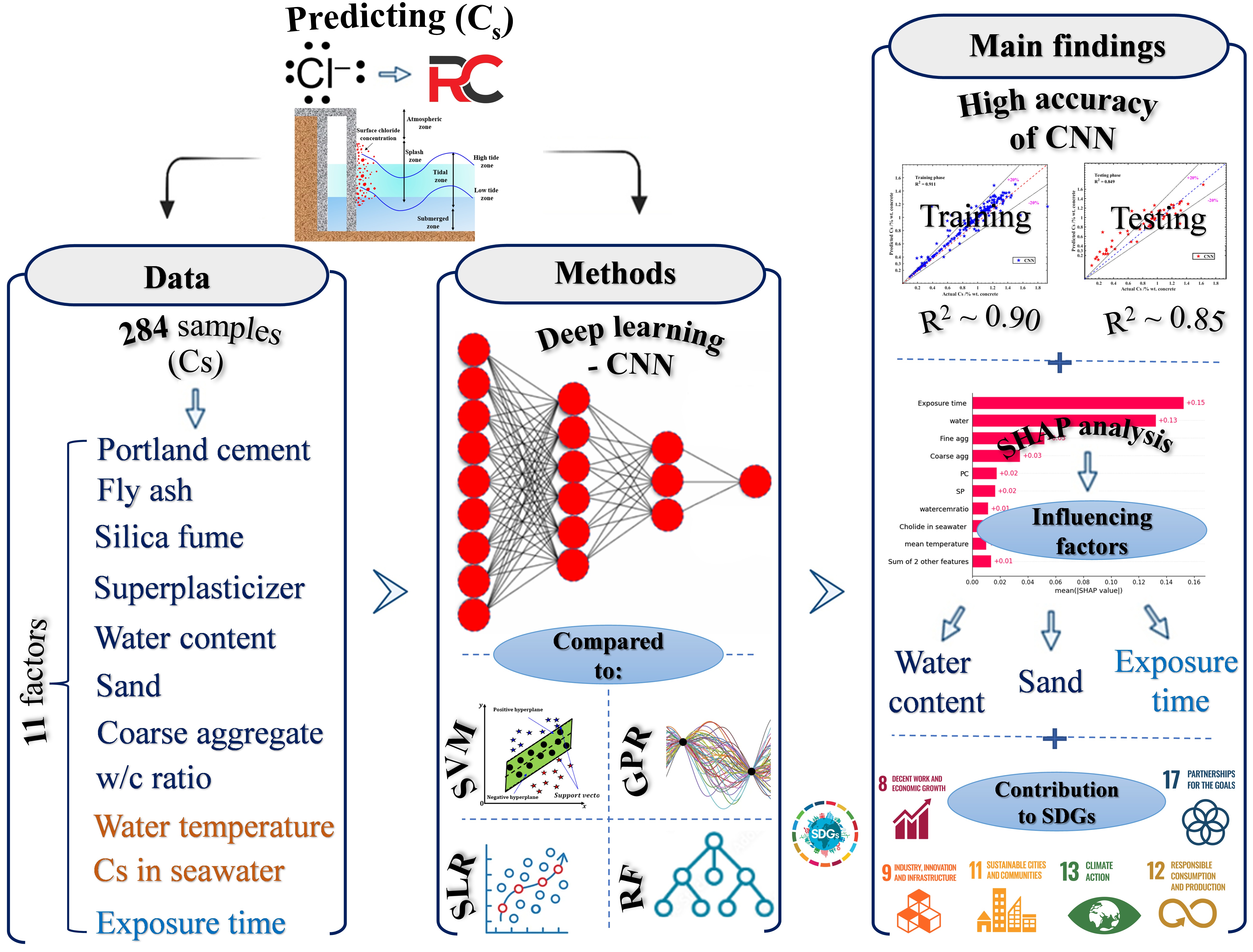

Supplement: Supplementary file 1 — Supplementary Figure [file 41598_2025_12035_MOESM1_ESM.jpg]
